# Vowels and Consonants in the Brain: Evidence from Magnetoencephalographic Studies on the N1m in Normal-Hearing Listeners

**DOI:** 10.3389/fpsyg.2016.01413

**Published:** 2016-09-22

**Authors:** Anna Dora Manca, Mirko Grimaldi

**Affiliations:** ^1^Dipartimento di Studi Umanistici, Centro di Ricerca Interdisciplinare sul Linguaggio, University of SalentoLecce, Italy; ^2^Laboratorio Diffuso di Ricerca Interdisciplinare Applicata alla MedicinaLecce, Italy

**Keywords:** magnetoencephalography, N1, vowels, consonants, auditory cortex, tonotopy, tonochrony, oscillatory rhythms

## Abstract

Speech sound perception is one of the most fascinating tasks performed by the human brain. It involves a mapping from continuous acoustic waveforms onto the discrete phonological units computed to store words in the mental lexicon. In this article, we review the magnetoencephalographic studies that have explored the timing and morphology of the N1m component to investigate how vowels and consonants are computed and represented within the auditory cortex. The neurons that are involved in the N1m act to construct a sensory memory of the stimulus due to spatially and temporally distributed activation patterns within the auditory cortex. Indeed, localization of auditory fields maps in animals and humans suggested two levels of sound coding, a tonotopy dimension for spectral properties and a tonochrony dimension for temporal properties of sounds. When the stimulus is a complex speech sound, tonotopy and tonochrony data may give important information to assess whether the speech sound parsing and decoding are generated by pure bottom-up reflection of acoustic differences or whether they are additionally affected by top-down processes related to phonological categories. Hints supporting pure bottom-up processing coexist with hints supporting top-down abstract phoneme representation. Actually, N1m data (amplitude, latency, source generators, and hemispheric distribution) are limited and do not help to disentangle the issue. The nature of these limitations is discussed. Moreover, neurophysiological studies on animals and neuroimaging studies on humans have been taken into consideration. We compare also the N1m findings with the investigation of the magnetic mismatch negativity (MMNm) component and with the analogous electrical components, the N1 and the MMN. We conclude that N1 seems more sensitive to capture lateralization and hierarchical processes than N1m, although the data are very preliminary. Finally, we suggest that MEG data should be integrated with EEG data in the light of the neural oscillations framework and we propose some concerns that should be addressed by future investigations if we want to closely line up language research with issues at the core of the functional brain mechanisms.

## Introduction

Making sense of speech contexts is a challenging task. The categorization of complex sounds requires the human brain to analyze the acoustic (phonetic) properties and perform computations integrating the analyzed properties into a perceptual (abstract) representation subjected to categorical (phonological) processes.

**Figure 1:**
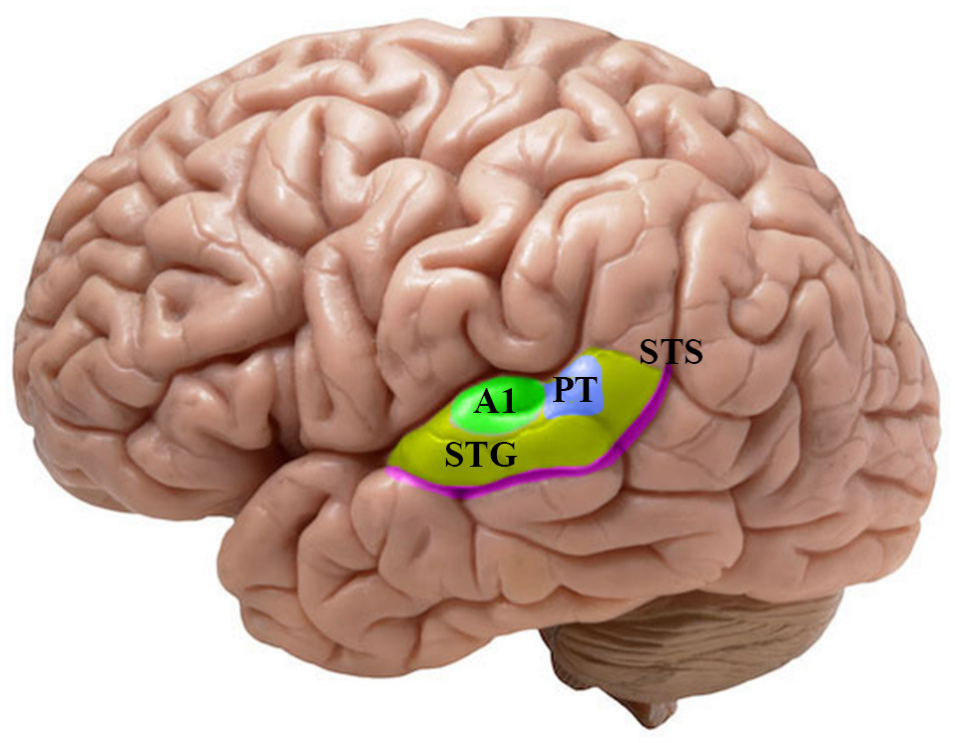
**Lateral view of the left hemisphere with a focus on the main human auditory areas housed in the supratemporal plane**. The colored patches show the different cortical fields which has been emphasized in the reviewed literature. Green: A1, primary auditory cortex in the Brodmann area (BA) 41. Indigo: PT, planum temporale. Yellow: STG, superior temporal gyrus in the BA 22. Purple: STS, superior temporal sulcus.

The neuroimaging investigations of the last 30 years have suggested a wide interrelated brain network for language processing (Price, 2012). The crucial area for the mapping of the acoustic-phonetic input signal into discrete mental representations is the auditory brain, which is the focus of our analysis (cf. Figure 1). The auditory areas are characterized by a layout that is highly specialized in analyzing different aspects of the signal: the primary auditory cortex (A1) seems engaged in the acoustic processing of the signal, while the superior temporal gyrus (STG) and the superior temporal sulcus (STS) work smoothly for encoding the acoustic patterns onto phonological features (Scott and Johnsrude, 2003; Santoro et al., 2014; for the speech perception and production link see Hickok and Poeppel, 2007; Rauschecker and Scott, 2009; Cheung et al., 2016). However, the localization and the lateralization of the structures engaged in the phonological encoding remain subjects of debate (McGettigan and Scott, 2012; Scott and McGettigan, 2013; Specht, 2013; Specht et al., 2014; Talavage et al., 2014; see also Cogan et al., 2014). Most importantly for the purpose of this work, the processing that leads speech sounds to be computed and represented within the auditory cortex is yet not fully understood. With the advent of the source localization techniques in the late 1970s, it became practical for electrophysiological and neuromagnetic investigations to evaluate the local organization and the response properties within the central auditory pathway (Romani et al., 1982). Thus, the analyses of timing and selective activation of the auditory cortex by speech sound stimulation permitted the acquisition of relevant knowledge on the neural basis of speech perception (Roberts et al., 2000; Poeppel, 2003; Boemio et al., 2005). The amount of data accumulated up to now demands a critical review of the findings concerning the spatiotemporal processing of speech sounds at the interface of linguistic and neurophysiological primitives (Poeppel and Embick, 2005; Grimaldi, 2012; cf. Section A Brief Look At Linguistic and Neurophysiological Primitives).

In this perspective, three techniques are widely used: (1) electroencephalography (EEG); (2) magnetoencephalography (MEG), and (3) electrocorticography (ECoG), an invasive approach used prevalently in clinical contexts where pre-surgical evaluation of cognitive processes is needed (Poeppel and Hickok, 2015). MEG is one of the most powerful non-invasive tools used in auditory neuroscience. Like EEG, MEG shows high temporal reliability, and because of its dipolar nature and its precise source localization, it is preferred to EEG (Roberts et al., 2000). EEG and MEG research into language processing is based on event-related potentials (ERPs) and event-related magnetic fields (ERMFs) recorded while the subjects are performing a task (cf. Section MEG in Short). They are characterized by a specific pattern called waveform (or component) normally grouped into an overall average for each subject that shows typical polarity (negative or positive), timing in milliseconds (ms) after the event (latency) and scalp distribution (Luck, 2005). In response to sound stimuli in particular, the deflections in this waveform are known as auditory evoked fields or AEFs (the equivalent of the EEG auditory evoked responses or AEPs).

The auditory components widely investigated are N1, with its magnetic counterpart N1m, and mismatch negativity (MMN), with its magnetic counterpart MMNm. N1/N1m is a negative peak between 70 and 150 ms after the onset of an auditory stimulus that appears to be involved in the basic processing of speech sounds in auditory cortices (Woods, 1995). It seems that the amplitudes and in particular the latencies of the N1/N1m are relevant markers reflecting the cortical encoding of acoustic features of incoming speech sounds. Also, the dipole location of the N1m responses along the auditory planes (cf. Section The N1m/N1 Generators for Vowels, Consonants, and Syllables) seems to be driven by the spectral properties that are linguistically salient: e.g., the F1/F2 ratio for vowels, or the place of articulation for consonants.

MMN/MNNm is a component temporally subsequent to the N1/N1m, automatically and preattentively elicited by an acoustic change or by a rule violation between 150 and 250 ms post-stimulus onset (Näätänen, 2001). Contrary to the N1/N1m, it is generated in a passive oddball paradigm, where subjects listen to frequent (standard) stimuli interspersed with infrequent (deviant) stimuli and attend to a secondary task (e.g., watching a silent movie). MMN/MMNm is visible by subtracting standard responses from deviant responses to the same acoustic stimuli and its amplitude seems to be directly correlated with the discriminability of the two stimuli involving both acoustic change-detection processes and phoneme-specific processes (Näätänen et al., 2007, 2011; Sussman et al., 2013). Thus, this component has been exploited to investigate (i) the categorical representation of phonemes in the subjects' mother tongue (e.g., Näätänen et al., 1997); (ii) if the acoustic signal is mapped onto lexical representations through different levels of featural representation; in this case, N1m and MMNm have also been used together (Scharinger et al., 2011b, 2012) (cf. Section Summary), and (iii) if phonemic representation may eventually develop during second language acquisition (Zhang and Wang, 2007; Moreno et al., 2008; Grimaldi et al., 2014, and the literature there discussed).

The approach discussed above opens an important window on the time course and the neural basis of speech processing. Indeed, more than 100 years after the initial discovery of EEG activity, researchers are turning back to reconsider another aspect of event-related EEG activity, that is, the fluctuations in rhythmic, oscillatory activity (Giraud and Poeppel, 2012). It has been argued that ERP does not simply emerge from evoked, latency–fixed polarity responses that are additive to and independent of ongoing EEG: instead, evidence suggests that early ERP components are generated by a superposition of ongoing EEG oscillations that reset their phases in response to sensory input (i.e., the stimuli generating cognitive activities; Sauseng et al., 2007). In brief, contrary to phase-locked responses (ERPs), non-phase-locked responses predominantly reflect the extent to which the underlying neuronal activity synchronizes. Since synchronization and desynchronization are indicative of the coupling and uncoupling of functional networks, respectively, it follows that event-related, non-phase-locked oscillatory EEG responses may provide an alternative way to study the functional network of the linguistic brain (Bastiaansen et al., 2012; Weisz and Obleser, 2014).

Here we review the contribution of the N1m component in understanding speech auditory processing. In particular, we are interested to show to what extent the data obtained from MEG recordings in normal-hearing subjects prove that the continuous acoustic flow characterizing speech sounds is decoded onto abstract phonemic representations. After describing the linguistic and neurophysiological principles that guide this field of research, we introduce the readers to the basics of MEG and N1m principles compared to MMNm principles. We then critically discuss the amplitude, latency, and source generators, and the results concerning the hemispheric lateralization processes for vowels, consonants (and syllables where applicable). In parallel, we compare these findings to those obtained from EEG studies demonstrating that the available MEG data are limited for supporting the view of abstract phoneme representations. The nature of these limitations is also discussed. We suggest that MEG and EEG research should be better integrated because EEG seems more sensitive to capture the hierarchy of processing and the lateralization processes of signals. Finally, we discuss this issue in the light of the neural oscillations framework proposing some important concerns which will should be the subject of future investigation into the field.

## A brief look at linguistic and neurophysiological primitives

### Linguistic primitives

We began this work by stressing the classical issues for linguistic theory and by placing them within a neurobiological perspective (cf. Section Introduction). Three key questions emerge: (i) what are the distinctive elements that characterize language sound systems? (ii) how are they acquired? (iii) how are they mentally represented? These questions were raised by the empirical observation that children acquire language by decoding the stream of continuously varying sounds to which they are exposed onto discrete representations and, ultimately, into meaning. So, the physical attributes of the signal need to be transformed into abstract mental representations. This has led linguists to distinguish between a *phonetic* level and a *phonological* level of analysis, which presuppose different kinds of representations: a conversion from acoustic-phonetics patterns onto phonological (abstract) representations to generate lexical and syntactic representations.

**Figure 2:**
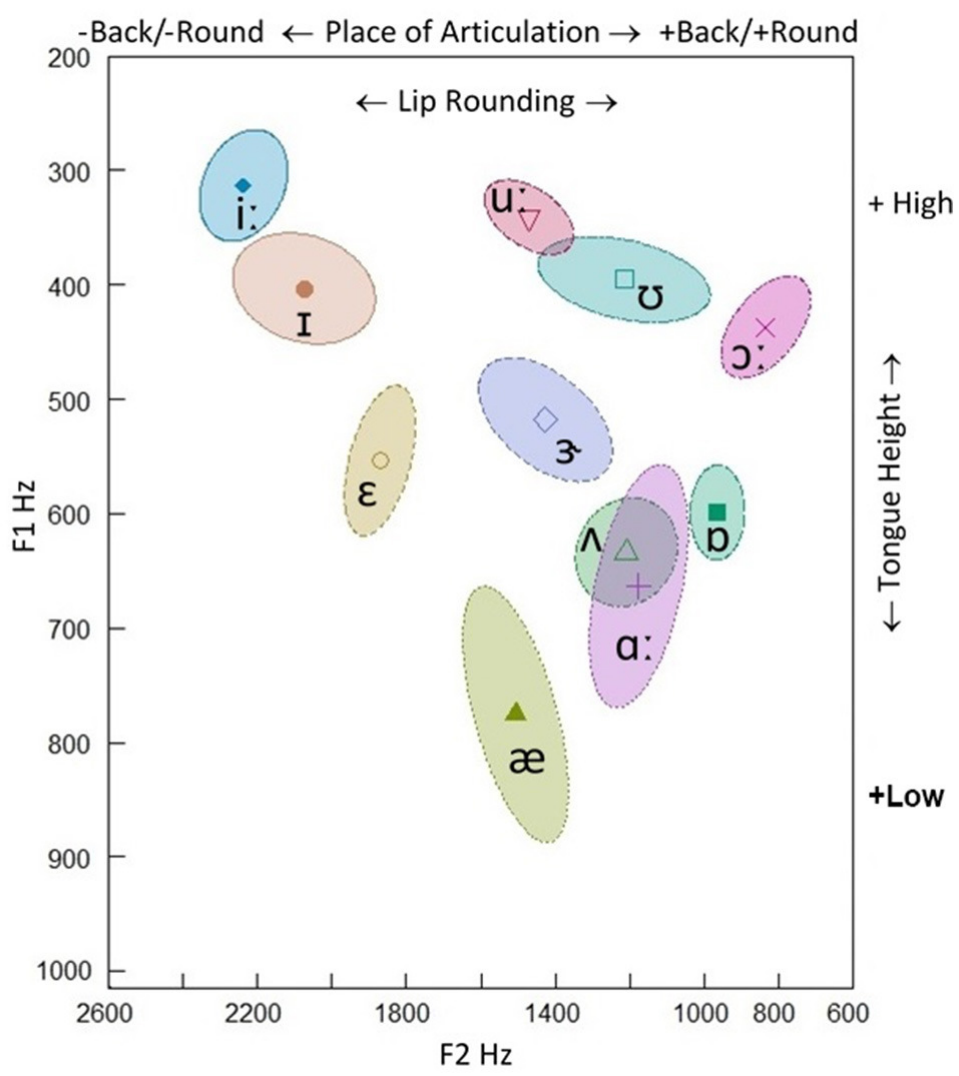
**F1-F2 Hz scatterplot of the stressed British English vowels produced by a native 50-year-old male speaker (our recording)**. 68.27% confidence ellipse corresponding to ±1 standard deviation from the bivariate mean (the symbol within the ellipse indicates the mean formant value). F1 is inversely correlated with articulatory tongue height (+high/−high), while F2 reflects place of articulation in the horizontal (−back/+back and −round/+round) dimension.

In the late 1950s, a model of speech recognition was developed to reconcile (to some extent at least) this separation between the two levels of representation: i.e., the *analysis by synthesis* framework (Stevens and Halle, 1967; Stevens, 2002). The analysis by synthesis theory assumes top-down processes in which potential signal patterns are internally generated (synthesized) and compared to the incoming signal; thus, perceptual analysis crucially contains a step of synthetically generating candidate representations (a form of hypothesis-and-test model). The model proceeds from the assumption that cues from the input signal trigger guesses about “landmarks” that serve to identify phoneme boundaries: as a consequence, the internal synthesis of potential phonemes is compared to the input sequence. Thus, landmarks are intrinsic to the signal and provide evidence for different kinds of segments (vowels, glides, and consonants): e.g., a peak in low-frequency amplitude for a vowel, a minimum in low-frequency amplitude, without acoustic discontinuities, for a glide, and two acoustic discontinuities for a consonant, one of which occurs at the consonant closure and one at the consonant release (Stevens, 2002: p. 1873). For example, vowels may be classified on the basis on the first two formant peaks (F1, F2) on the spectral envelopes (Peterson and Barney, 1952). The F1 is inversely correlated with articulatory tongue height, while the F2 (but also F3) reflects the place of articulation (PoA) along the horizontal (front-back and unrounded-rounded) dimension. The major features for vowels are the features specifying the position of the tongue body and lip rounding: the features [±high], [±low], [±back] and [±round] (Figure 2). In consonants, beyond formants, additional physical parameters are essential for discriminative performance: e.g., formant transitions, energy bursts, and the vibrations of the vocal chords occurring before and during the consonant burst.

So, speech-mapping principles are determined by acoustic-articulatory properties that are affected by top-down features based on abstract properties relating to articulator positions that yield a discrete representation of the speech stream in terms of a sequence of segments (Poeppel et al., 2008). Each segment is described by a set (or bundle) of binary distinctive features that represent the abstract linking between articulatory plans and acoustic outputs (Halle, 2002). They are characterized by polar oppositions and are the primitives for phonological computation and representation, as the smallest units that contrastively, change the meaning of a single word (e.g., [k] and [r] in [‘kæt] *cat* vs. [‘ræt] *rat*). Words are, then, represented as a series of segments each of which is a bundle of distinctive features that indicate the acoustic-articulatory configuration underlying the phonological segments (Poeppel et al., 2008: p. 1072) This model was recently confirmed in the results of a MEG study (cf. also Section Neurophysiological Primitives). Kuhl et al. (2014) showed that 7 month-old infants activate auditory and motor brain areas similarly for native and non-native sounds, whereas 11–12 month-old infants activate auditory brain areas for native sounds and motor brain areas for non-native sounds (matching the adult pattern). These data clearly suggest that the auditory analysis of speech is coupled with the synthesis of the motor plans that are necessary to produce the speech signal.

Nevertheless, the variability of acoustic realizations of individual speech sounds, the effects in co-articulation, and the phonological context do not allow a direct and simple mapping between acoustic and linguistic features; thus, in tracing the functioning of the auditory activity in speech processing, the effects of these and further variables need to be taken into account.

### Neurophysiological primitives

Speech perception is a very early capability. Sensibility to speech input begins in the womb, as the fetuses become attuned to a variety of features of the surrounding auditory environment. As recently showed by infant MMN data (Partanen et al., 2013), this capability may play an important role in the early speech discrimination of newborns by facilitating learning to decode the incoming speech signal into discrete units by means of probabilistic and statistical operations computed by the brain on the acoustic signal (Kuhl, 2004). In the first year of life, a clear perceptual transition from all the possible (universal) learning options to language-specific learning options emerges. Before 6–8 months of age, infants are able to discriminate all the contrasts phonetically relevant in any of the world's languages; by 12 months their discrimination sensitivity is warped by native phonemes while the perceptual sensitivity for non-native phonemes gradually declines (Werker and Tees, 2005; Kuhl et al., 2006). However, a recent gamma oscillations study showed that this cerebral reorganization around native categories is already formed at 6 months of age (Ortiz-Mantilla et al., 2013) and may reflect a continuous process of neural commitment toward the first language and a gradual decrease in neural plasticity to acquire another language (Zhang and Wang, 2007). The “perceptual magnet” formed around native sounds does not however impede the establishment of new phonetic categories during second language acquisition, also in adult learners (Flege, 1995; Best and Tyler, 2007). Nonetheless, the extent of success may depend on numerous variables: i.e., age of L2 learning, length of residence in a second language-speaking country, formal instruction, amount of native language use, quality and quantity of second language stimuli (Piske et al., 2001).

The reshaping of the perceptual space in infants according to the acoustic-phonetic properties of the mother tongue implies that constant computational processes on the signal are encoded online into abstract discrete representations of sounds (Giraud and Poeppel, 2012). A natural hypothesis is that the acoustic-phonetic structures map directly onto clusters of neurons within the auditory cortex thanks to the specific sensitivity of nerve cells to the spectral properties of sounds: i.e., the so-called *tonotopic (phonemotopy) principle* (Romani et al., 1982). This place coding of acoustic frequencies is ensured by the selective activation of the cochlear neurons regularly positioned along the basilar membrane (Moerel et al., 2014; Saenz and Langers, 2014). Then, the neural signals emitted by cochlear neurons are transmitted in the brainstem and preserved up to the auditory cortex from the A1 to the STG and the STS (Wessinger et al., 1997; Talavage et al., 2004; Da Costa et al., 2011; cf. Figure 1); while pre-cortical processing seems to be common to all sounds, speech-specificity appears to arise at the cortex (Scott and Johnsrude, 2003). Furthermore, it has been suggested that neural oscillations (in particular high gamma field potentials) constitute a possible mechanism for spatiotemporally discretizing speech sounds in the STG regions (Mesgarani et al., 2014). Like retinotopy in vision (Palmer, 1999), tonotopy is one of the most accepted models of cortical organization of the auditory pathway (Moerel et al., 2014) as also showed by studies on animals (Kaas and Hackett, 2000; Rauschecker and Tian, 2000; Mesgarani et al., 2008) and it represents the fundamental rule of sound processing (Schreiner et al., 2000). In addition to the topographical separation of sounds of different frequencies, it has been suggested that latency may be a supplementary dimension for object encoding in the auditory system. Roberts and Poeppel (1996) demonstrated that there is a frequency dependence of latencies separate from stimulus intensity (see also Roberts et al., 1998, 2000, 2004). Furthermore, recent animal data has shown that the precision of temporally based neural representations declines from periphery to the cortical regions entailing different encoding strategies for slow and fast acoustic modulations (Wang, 2007). Thus, the temporal code may represent the ability of some pools of neurons to discharge at a particular phase of the structure of sounds (Zatorre and Belin, 2001; Boemio et al., 2005). This temporal mechanism of auditory encoding is known as the *tonochrony* principle. That is, the latency of auditory evoked components appears to be sensitive to some stimulus properties; this suggests that the mechanism of tonochronic encoding might augment or supplement the tonotopic strategy in the frequency range critical to human speech (*phonemochrony*) (Roberts et al., 2000). However, the nature of this temporal specification for speech sounds remains unclear.

### Summary

In brief, a long-standing proposal of linguistic theory is that the relevant representational primitives are not single segments, phonemes, but rather smaller units of which segments are composed: i.e., distinctive features. Distinctive features are intrinsic to the speech perception and production dimensions and therefore they are founded on neurobiological principles. Two neurophysiological primitives seem to give account of the strategies that the auditory system uses to compute and represent sounds: a place code (*phonemotopy*) for spectral information and a temporal code (*phonemochrony*) for temporal information. The place code refers to the specialization of the auditory tissues to process the spectral frequencies of stimuli, whereas the temporal code relates to specific timing response of neurons to distinct features of sounds. What we are going to do in the following sections is to try to understand if the available evidences can legitimately be used to coherently link linguistic primitives with neurophysiological primitives for what concerns the computation and representation of speech sounds.

## The MEG and the N1/N1m component

### MEG in short

**Figure 3:**
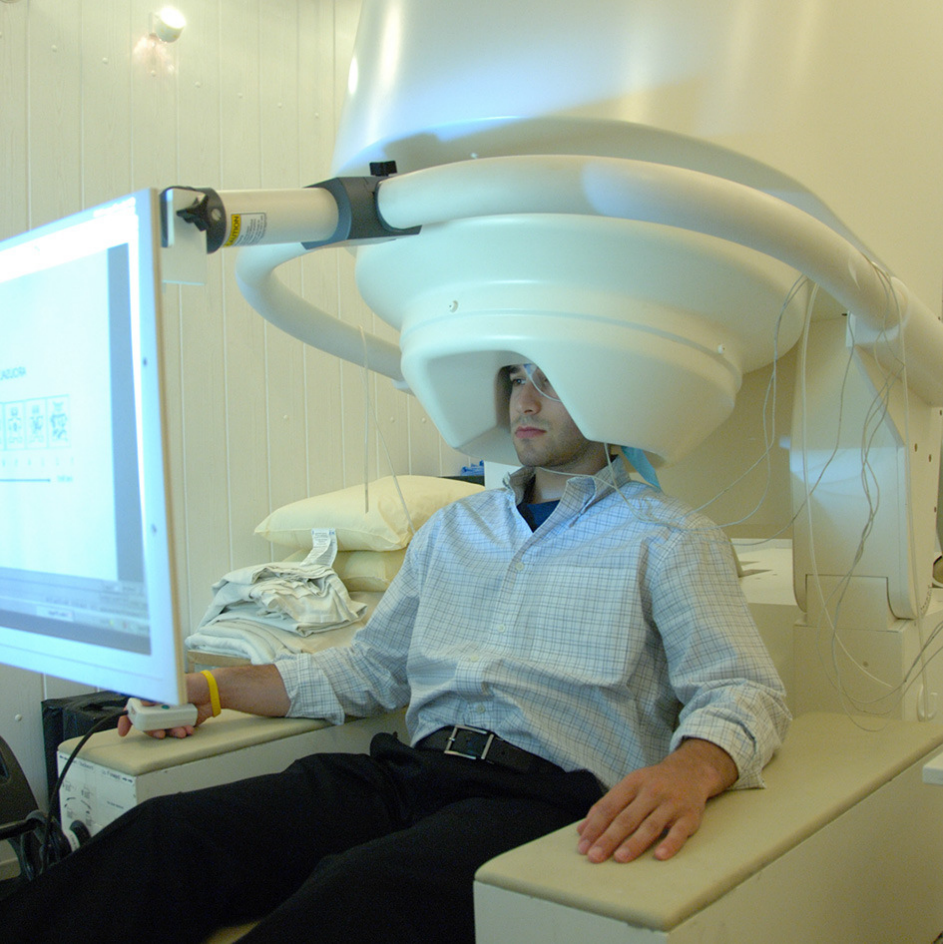
**MEG setup**. The recording systems is situated in a magnetically shielded room maintaining an electromagnetically quiet recording environment. Subjects are positioned either sitting or supine with their heads in the recording helmet that covers as much of the skull/brain as possible. The detectors embedded in the helmet work as high-gain low-noise amplifier of the magnetic field elicited by the neuronal activity (Poeppel and Hickok, 2015). A 4D Neuroimaging MEG system that uses the magnetometer sensors is showed. From https://en.wikipedia.org/wiki/Magnetoencephalography.

MEG detects the activity at the cortex by measuring small magnetic fields of primary and volume currents with particular multi-channel detectors (Figure 3): i.e., the superconducting quantum interference devices (SQUIDs) positioned over the scalp (Gross et al., 2013; Supek and Aine, 2014). Recent neuromagnetometers contain helmet-shaped arrays of more than 300 SQUID sensors using magnetometer (consisting of a single superconducting coil) or gradiometer (consisting of two oppositely wound coils) (Hansen et al., 2010). The magnetometer is most sensitive to source currents a few centimeters outside of the loop (and may detect deeper sources) but can also pick up environmental noise, whereas the gradiometer yields the maximum signal directly above the source current, thus markedly facilitating the sensor-based estimation of source configuration as the first step of source analysis. Modern MEG systems are equipped with both magnetometers and gradiometers allowing simultaneously MEG and EEG recordings. Also, the recent introduction of SQUID-based low-field (microtesla) MRI has created a new means of integrating MEG and MRI information within the same recording, raising expectations for improved spatiotemporal accuracy of the measured signals (Hari and Salmelin, 2012).

From the ongoing brain activity is possible to extract distinct neuronal responses—ERPs and ERMFs components—by time locking the brain signals to the onset of any external event. These responses reflect the summated activity of a large number of neurons firing synchronously and are commonly used for tracking the neuronal phenomena of cognitive processes. In this vein, the AEFs and AEPs are intended as valid signatures of the organizing principles the auditory and speech processing.

**Figure 4:**
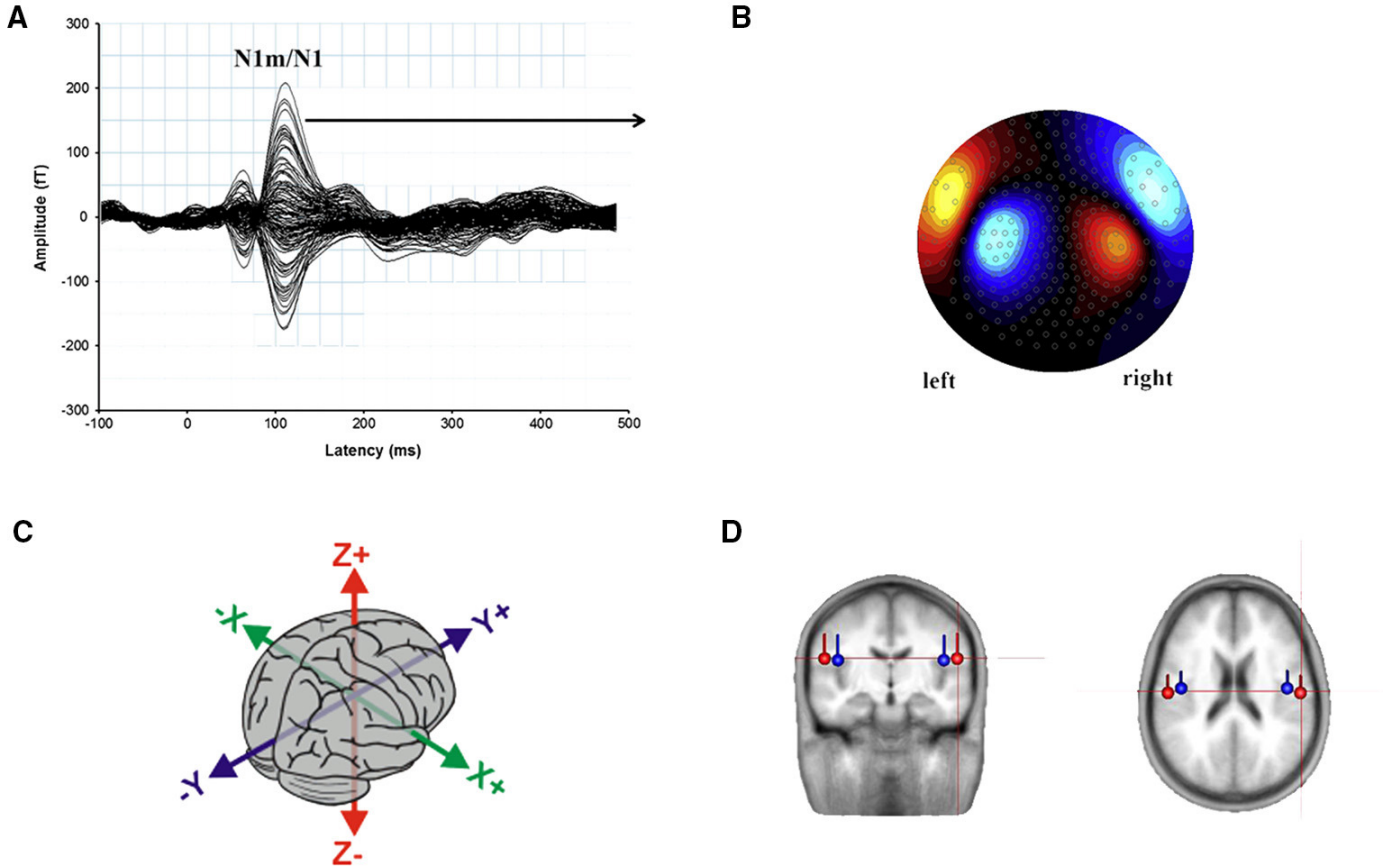
**(A)** Representation of the auditory N1m/N1 wave evoked from 275 channels to a kHz tone presented to the right ear. The peak at 100 ms post-stimulus onset, measured in femto-Tesla (fT) is evidenced. **(B)** The topographic map represents the direction and amplitude of the response at the N1m peak. Adapted from Sereda et al. (2013: p. 302). **(C)** The 3D space within the brain along the classical Talairach coordinates: The direction of *x* axis is from left to right, that of *y* axis to the front, and the *z* axis thus points up. **(D)** Average location and orientation of the equivalent current dipole sources fitted in the bilateral auditory cortical areas. Adapted from Cirelli et al. (2014).

As the signals measured on the scalp surface do not directly indicate the location of the active neurons in the brain, when interpreting MEG (and EEG) data, one has to solve the so-called the *inverse problem*, i.e., the deduction of the source currents responsible for the externally measured fields (Mosher et al., 1999; Hallez et al., 2007). Although there is no single solution, with appropriate constraints, it is possible to simulate the neural activity by means of a dipolar model (Malmivuo et al., 1997). Dipoles are created by post-synaptic potentials of many single neurons oriented in the same direction and firing synchronously in response to the same event. Under stimulation, the dipoles from the individual neurons sum solving in a single equivalent current dipole (ECD) that seems to be the best approximation of ARFs (and AEPs) observed by sensors on the scalp. Location, orientation, and magnitude of the assumed ECDs provide information about the behavior of the activity under investigation (Luck, 2005). The ECD can be described as a point located in a 3D space within the brain along the classical Talairach coordinates that represent the center of simultaneously active neural sources (Wendel et al., 2009; Sanei and Chambers, 2013): i.e., *x* (lateral-medial), *y* (anterior-posterior), and z (inferior-superior) axes (Figure 4C).

It is important to keep in mind that even MEG and EEG have similar temporal resolutions, they have a different sensitivity to the dipole orientation: MEG is insensitive to radial dipoles whereas EEG is equally sensitive to radially and tangentially oriented sources (Cohen and Halgren, 2003; Ahlfors et al., 2010). As we will show, this means that, potentially, both techniques are to be considered as complementary in terms of the neurophysiological information they provide (Sections The N1/N1m Wave and its MMN/MMNm Counterpart and In the Left, in the Right or in Both Sides?).

### The N1/N1m wave and its MMN/MMNm counterpart

N1/N1m is the most prominent of the early auditory responses and it typically peaks at around 100 ms after the onset of a new stimulus showing maximum amplitude over the fronto-central areas of the scalp (Figures 4A,B). It is assumed that N1 reflects the basic operations of construction of perceptual representations (Näätänen and Picton, 1987). Pioneering N1m studies on non-speech stimuli showed that the N1 latency seems related to the (perceived) pitch and other spectrally prominent frequencies, whereas the N1 amplitude may reflect stimulus intensity (Elberling et al., 1981; Roberts and Poeppel, 1996; Roberts et al., 1998, 2000). Converging evidence suggests that subjective factors such as gender (Obleser et al., 2001), age of the experimental subjects (Näätänen and Picton, 1987), and particular experimental manipulations have modulatory effects reflecting in a substantial alteration of N1m. For example, the stimulation rate affects the amplitudes as a function of the inter-stimulus interval (ISI): with very short ISIs (<300 ms) N1m becomes minute (Hari et al., 1982) at randomly varying ISIs ranging for example from 100 to 1000 ms, amplitude significantly enhances (Wang et al., 2008) whereas it does not change for ISIs longer than 10 s (Ritter et al., 1968). Again, the amplitude decrement was found to be dependent on stimulus intensity (Zhang et al., 2009). Also, it has been shown that increases in stimulus rise-time generate long N1m latencies and small N1m amplitudes in humans and animals for pure tones or tone bursts (Kodera et al., 1979; Biermann and Heil, 2000). However, Grimaldi et al. (in press) showed that non-controlled rise times for speech sounds (natural vowels) do not affect the N1 latencies, but rather, they seem to be modulated by distinctive features (cf. Section Latency for Vowels, Consonants, and Syllables). On the contrary, it seems that the ramp of fall-time does not influence the N1m behavior, as it is only sensitive to temporal changes in the first ~40 ms of the signal (Gage et al., 2006).

Crucially, the scalp distribution of the auditory N1/N1m, and the effects of cerebral lesions on the N1/N1m responses to clicks, noise, bursts, and tones suggest at least three distinct N1 sub-components (Näätänen and Picton, 1987: p. 411). The first N1 sub-component is maximally recorded from the fronto-central scalp, peaks between 85 and 110 ms and is generated by tangentially orientated currents in both A1 (Vaughan and Ritter, 1970; Hari et al., 1980; Wood and Wolpaw, 1982); the second sub-component is detectable approximately at 150 ms in the mid temporal scalp regions and is generated by radially oriented neuronal sources in STG (Wolpaw and Penry, 1975; Picton et al., 1978; Wood and Wolpaw, 1982), and the third sub-component is a negative wave at the vertex at 100 ms whose generators are not known. Notwithstanding this peculiarity, the N1m underlying sources are commonly modeled by a single dipole in each hemisphere whose location seems to be dependent on the stimulus spectral frequencies (Elberling et al., 1981; Romani et al., 1982; Pantev et al., 1988, 1995; Tiitinen et al., 1993). The cortical origins were in primary auditory areas, at the lower bank of the lateral sulcus (Pantev et al., 1995; Diesch et al., 1996) and more recently, it has also been shown that the N1m might have source generators in the STG and in the planum temporale (Inui et al., 2006) suggesting its crucial role for the final (May and Tiitinen, 2010) rather than for the initial stages (Näätänen and Picton, 1987) of sensorial data processing (Figures 4C,D). The above-cited studies revealed a certain cortical selectivity to sound properties as reflected in the ECD behavior within the auditory brain. For example, they found that the dipoles to high-frequency tones are medial to the sources for low frequency tones. Also, it seems that the tonotopic gradient for pure-tone stimuli of different frequency runs approximately from inferior to superior axis, while the periodotopic gradient for harmonic stimuli of different F0 runs approximately from anterior to posterior locations (Langner et al., 1997: p. 672). As we will better see in the following sections, recent researches found hints for a separate, specific, and orderly representation of speech sounds in the auditory cortex suggesting that the locations of the N1/N1m dipoles may reflect a categorical distinction of speech sounds (phonemotopy) based on their resonance (formant) frequencies, in accordance with the analysis by synthesis model. These findings contrast with the results indicating identical processing of speech sounds and tones (Lawson and Gaillard, 1981; Woods and Elmasian, 1986).

Unfortunately, as noted in the previous Section, the magnetic recordings are relatively blind to radially oriented dipoles: thus, the relationship between the N1 events and the stimulus parameters cannot be fully explained by this kind of approach.

Are there specific motivations to choose between N1/N1m and MMN/MMNm in studying the auditory processing of speech sounds? The reasons to prefer one or the other component are probably related to theoretical and methodological issues. The evidence up to now suggests that, although N1m and MMNm may overlap, they reflect distinct cerebral processes. The N1m processes are associated with the nature of the stimulus itself, while the MMNm is associated with stimulus comparison or discrepancy (Näätänen and Picton, 1987). Thus, the N1m wave reflects the amount of neuronal activity occurring to trace a sequence of stimuli whereas the MMNm wave reflects the comparison between two consecutive stimuli (cf. also Picton et al., 2000; Winkler, 2007). The neurons involved in the N1m component may act to call attention to the availability of stimulus information, to read out sensory information from the auditory cortex (Näätänen and Picton, 1987) and, importantly, to construct a sensory memory of the stimulus within the auditory cortex (Näätänen et al., 2001). So, when the stimulus is a complex speech sound, it becomes reasonable to explore the N1m event in order to assess whether the spectrotemporal encoding of the signal properties is generated by a pure bottom-up reflection of acoustic differences between sounds (e.g., vowels and consonants) and whether it is additionally warped by linguistic categories. At the methodological level, we should underline that N1/N1m is elicited by sequences of auditory stimuli randomly presented: thus, observing the N1 modulations might actually reveal to what extent they mirror an underlying percept or correlate with the acoustic properties of stimuli. On the contrary, MMN/MMNm is elicited by using an oddball paradigm where only couples of stimuli can be presented as standard or deviant (although recently a multi-feature paradigm has been developed), which enables the recording of several mismatch negativities to phonetic and acoustic changes (Näätänen et al., 2004; Pakarinen et al., 2009; Partanen et al., 2011). In other words, N1/N1m seems more suitable than MMN to test whether and how the neural coding schemes (e.g., tonotopy and tonochrony) are used as the representations to be encoded become more complex and speech-specific; MMN/MMNm seems to be appropriate to investigate the speech-specific properties of peculiar phonetic categories within language systems, the development of such categories, and their representational processes during second language acquisition (Dehaene-Lambertz et al., 2000). Furthermore, the MMN/MMNm oddball paradigm seems very promising to investigate the nature of lexical access (Shtyrov and Pulvermüller, 2002a,b; Assadollahi and Pulvermüller, 2003) and certain aspects of syntactic processing (Shtyrov et al., 2003; Pulvermüller and Shtyrov, 2006). However, designing MMN/MMNm experiments for directly testing the status of phonological patterns, *per se*, remains a challenging task (Monahan et al., 2013: p. 248).

## The N1m/N1 amplitudes, latencies, and source generators

### Amplitudes for vowels and consonants

Initial MEG investigations showed that the acoustic features for speech and non-speech stimuli affected the amplitude responses setting vowels apart from tones or bursts (Eulitz et al., 1995; Diesch et al., 1996; Poeppel et al., 1997); for the tonal vs. speech stimuli, for example, N1/N1m amplitudes were significantly large (Swink and Stuart, 2012). However, the initial works with synthetic vowels given no indication of different underlying neural representations of speech sounds. Eulitz et al. (1995), using the synthetic vowels [a, æ, u, i, œ] and Diesch et al. (1996), testing the N1m responses to long (600 ms) and short (200 ms) synthetic vowels [a, æ, u, i] did not find vowel differences. Also Poeppel et al. (1997) using female and male synthetic vowels [a, i, u] at two different fundamental frequencies did not reveal significant effects of the phoneme type either (see Table 1).

**Table 1:** **Results for amplitude and latency of the stimuli (vowels and consonants) applied in the N1m/N1 studies**.

**References**	**Sj**	**Language**	**Stimuli**	**Amplitude**	**Latency**
Eulitz et al., 1995	11	German synthetic	[a, i, u, æ, œ] 1 kHz burst	–	–
Diesch et al., 1996	11	Synthetic	[a, æ, u, ø, i] 1 kHz sine tone	–	[a], [æ] < [u]
Poeppel et al., 1997	6	Synthetic	[a, i, u] 500 Hz pure tone	–	[a] < [i], [u]
Mäkelä et al., 2003	10	Finnish semi-synth.	[a, o, u]	–	–
Obleser et al., 2003a	12	German synthetic	[a, e, i]	[a] < [i, e]	[a] < [i], [e]
Eulitz et al., 2004	1	Synthetic	[a, e, i]	–	[a] < [e]—left hemisphere [e] < [i]—right hemisphere
Obleser et al., 2004a	21	German natural	[ø, o]	[ø] > [o]	[o] > [ø]
Obleser et al., 2004b	20	German natural	[a, e, i, ø, y, o, u]	–	[o], [u]—the latest peaks [i] > [e] [y]-[ø] > [i]-[e]
Scharinger et al., 2011a	12	Turkish natural	[i. y, ɛ, ɑ, œ, ɔ ɯ, u]	[u] > [ɑ] [u] > [y] [i] > [ɯ]	[a] < [i] [u] > [ɯ]
Scharinger et al., 2012	14	American English natural	[æ. ɛ, i]	[ɛ] (as deviant preceded by the standard [æ] > [ɛ] (as standard)	–
Grimaldi et al., in press	11	SI natural	[i, ɛ, ɑ, ɔ, u]	[i, u] > [ɑ, ɔ, ɛ]	[ɑ, ɔ, ɛ] > [i, u]
Kuriki et al., 1995	8	Synthetic	[na], [ka], [ha], [a]	–	[ka], [ha] < [a] [na]—the longest peak
Poeppel et al., 1996	6	Synthetic	[bæ, pæ, dæ, tæ]	–	[bæ, pæ, dæ, tæ] > left hemisphere in the discrimination task
Gage et al., 1998			[b], [p], [d], [t], [g], [k], [f], [r], [m], [r], [s]	([b], [p], [d], [t], [g], [k]) > ([f], [r], [m], [r], [s])	([f], [r], [m], [r], [s]) > right hemisphere
Gage et al., 2002	6	German	[ba, [da], [ga]	–	[ba] > [da], [ga]—right hemisphere
Obleser et al., 2003b	22	Natural German	[bø], [dø], [gø] [bo], [do], [go]	–	[go] > than others
Obleser et al., 2006	19	Natural German	[d], [t], [g], [k]	([d], [g]) > ([t], [k])	([d], [t]) < ([g], [k]) ([d], [g]) > ([t], [k])
Scharinger et al., 2011b, Experiment 1	13	American English natural	[aja], [awa]	–	[awa] < [aja] (as deviants)
Scharinger et al., 2011b, Experiment 2	15	American English natural	[ava] [aʒa]	–	[aʒa] < [ava]
Scharinger et al., 2011b, Combined analysis Experiment 1 and 2			[aja], [awa], [ava], [aʒa]	[awa], [aʒa] > [aja], [ava]	–

*The labeled Sj indicates the number of subjects that were submitted to statistical analysis. The column labeled Language reports the variety of languages and the type of stimuli used in the experimental protocols. The symbol (–) means that the results did not reach a statistically significant level or that they were not reported. All studies listed report results from both hemispheres, unless otherwise noted*.

**Figure 5:**
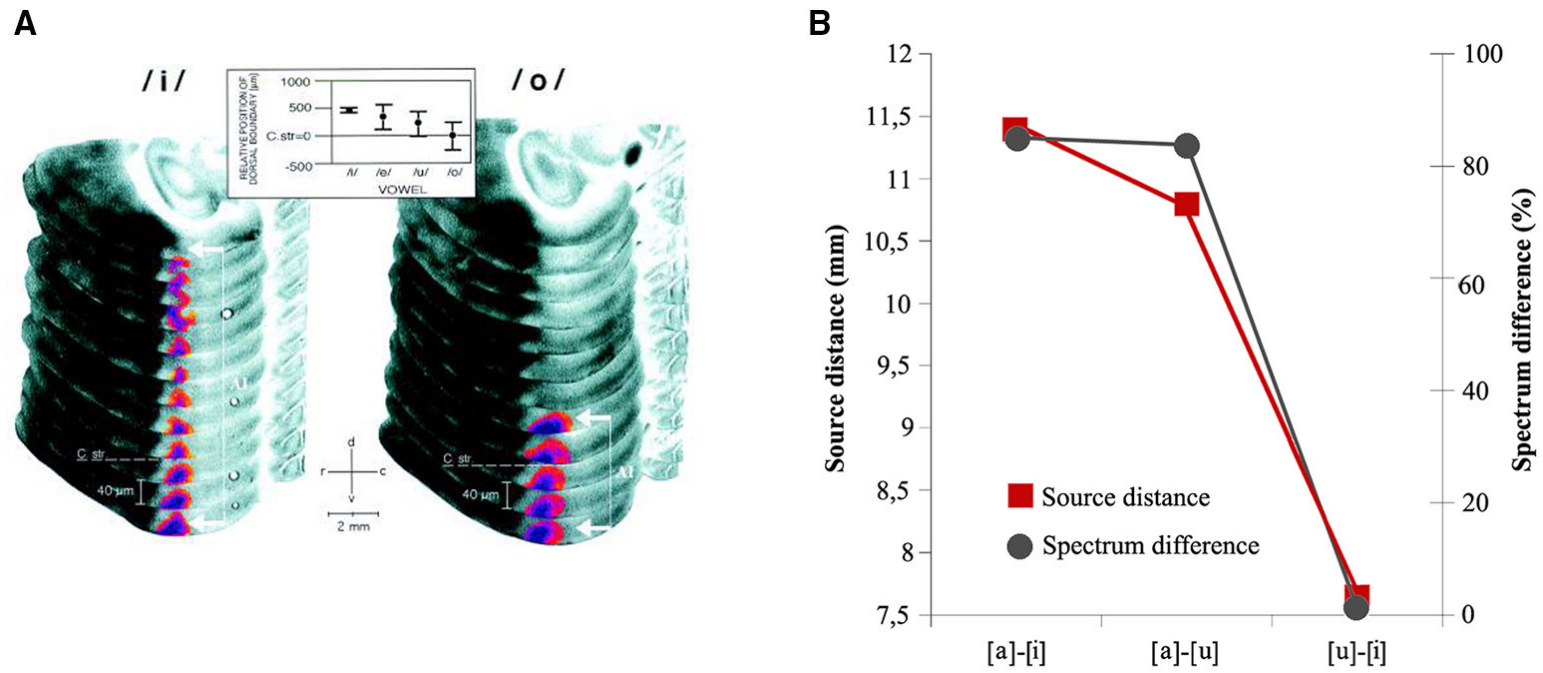
**(A)** FDG (2-Fluoro-2-Deoxy-D-[^14^C(U) Glucose) reconstruction of the activity patterns in left dorsal auditory cortex evoked by vowels [i] and [o] in Gerbils. Vowel representation in field A1 appeared as a dorso-ventral stripe along the isofrequency axis and was highlighted by using a pseudo-color transformation relative to the optical density of the corpus callosum. Large formant distances F2-F1, as in [i], led to stripes that extended far dorsally (white bracket with arrows), whereas stripes obtained with small formant distances, as in [o] ended close to the dorso-ventral level of the roof of the corpus striatum (C str. dashed line). At the top, topographic representation of formant distance F2-F1 along the isofrequency axis in A1 of the vowel-evoked FDG that were used in the study. From Ohl and Scheich (1997: p. 9442). *Copyright (1997) National Academy of Sciences, U.S.A*. **(B)** Graphical Representation of the relationship between mean distances of the source locations of [a]–[i], [a]–[u], [u]–[i] vowel pairs—measured via an ECD models- and relative acoustic–phonetic dissimilarity (black line) measured as F2/F1 ratio. The representational centers of the N1m show relative distances that resemble a *F2*-*F1* vowel space and indicate a phonemotopic organization in the supratemporal plane. Adapted from Shestakova et al. (2004: p. 348).

Subsequent works focusing on vowels having maximal variation in terms of F2-F1 difference found significant vowel differences as a function of acoustic category. Diesch and Luce (1997, 2000) explored the cortical encoding of two-formant vowels and their decomposed formants. They found that the size of the activated cortical patch was larger for those vowels characterized by a F2–F1 distance of more than 2000 Hz (e.g., in [i]) and smaller for those vowels with a formant distance of about 500 Hz (e.g., in [o]). In other words, the cortical mapping of complex stimuli appeared to mirror the interactions of the extracted prominent peaks in the frequency spectrum. Several studies have replicated this finding. A relationship between the vowel properties and the N1m amplitude was observed by Obleser et al. (2003a). They explored the cortical representation of the three German vowels [a], [e] and [i] (where [a] and [i] span a great distance in the F2-F1 space and [e] has an intermediate position) and revealed a weaker N1m amplitude to [a] than [e] and [i]. Thus, vowels marked by close F2-F1 formants peaks elicited weaker amplitudes than vowels with large F2-F1 differences. Shestakova et al. (2004) obtained the same results for multiple exemplars of the Russian vowels [a, u, i] (150 exemplars for each category): again, the [i] vowel, with the largest inter-formant distance, showed larger N1m response than the [a], [u] vowels. The investigation by Mäkelä et al. (2003) with the semi-synthetic sustained Finnish vowels [a], [o] and [u] extended such data by showing inconsistent differences for the three vowels that are marked by equal F2-F1 differences (see Table 2). However, Eulitz et al. (2004), which used the same stimuli as Obleser et al. (2003a), did not reveal N1m modulations related to the inter-formant frequency values. Altogether, the N1m result is not new at all, even for auditory animal models. In fact, Ohl and Scheich (1997) found tonotopic activation in the low-frequency in Gerbils “cortex was dependent on the vowels” F2-F1 distance: the activated regions were small for vowels with neighboring F1 and F2 and large for vowels with a large inter-formant distance (Figure 5A). All of the studies discussed up to now, interpreted data at the light of the inhibition principle already hypothesized by Shamma (1985a,b) according to which there exists a vowel-specific reduction of neuronal activity that depends on the vowel formant distance F2-F1 and that may be topographically organized along isofrequency contours as discussed in Section The N1m/N1 Generators for Vowels, Consonants, and Syllables (Figure 5B).

**Table 2:** **Pitch (F0), Formant Frequency (F1, F2, F3 in Hz) and formant distance (F2-F1) values of the vowels used as stimuli in the reviewed studies**.

**References**	**Vow**.	**F0**	**F1**	**F2**	**F3**	**F2-F1**
Eulitz et al., 1995; Diesch et al., 1996	/i/		250	2700	3400	
Poeppel et al., 1997		m 100 f 200	M 280 f 310	M 2250 f 2790	M 2890 f 3310	
Obleser et al., 2003a; Eulitz et al., 2004		From 129 to 119	250	2700	3400	
Obleser et al., 2004b		Min 127 max 132	Min 267 max 287	Min 2048 max 2120	Min 2838 max 328	Min 198 max 125
Shestakova et al., 2004		From 129 to 119	370	2250	2800	
Scharinger et al., 2012		184.31	531.50	2239.90	3009.50	
Grimaldi et al., in press		294	2325	2764	2031	294
Obleser et al., 2003a; Eulitz et al., 2004	/e/	From 129 to 119	370	2250	2800	
Obleser et al., 2004b		Min 109 max 125	Min 302 max 322	Min 2055 max 2143	Max 2890 min 2711	Min 1741 max 1821
Scharinger et al., 2011a	/ɛ/		550	2100	2700	
Scharinger et al., 2012		177.19	801.00	2008.80	2895.80	
Grimaldi et al., in press		145	549	1880	2489	1330
Eulitz et al., 1995	/ae/		606	2077	2656	
Diesch et al., 1996			600	2080	2650	
Scharinger et al., 2012		171.25	1023.30	1760.60	2712.60	
Eulitz et al., 1995; Diesch et al., 1996	/a/		780	1250	2600	
Poeppel et al., 1997		M 100 f 200	M 710 f 850	M 1100 f 1220	M 2540 f 2810	
Mäkelä et al., 2003						330
Grimaldi et al., in press		140	794	1231	2528	418
Obleser et al., 2003a; Eulitz et al., 2004		From 129 to 119	780	1250	2600	
Obleser et al., 2004b		Min 103 max 113	Min 552 max 747	Min 1188 max 1224	Min 2663 max 3171	Min 442 max 641
Scharinger et al., 2011a		680	1200	2700	680	1200
Mäkelä et al., 2003	/o/					350
Obleser et al., 2004a		M 123 f 223	M 317 f 390	M 516 f 904	M 2601 f 2871	M 199 f 514
Obleser et al., 2004b		Min 109 max 1125	Min 293 max 346	Min 471 max 609	Min 2481 max 2688	Min 131 max 303
Scharinger et al., 2011a		500	900	3000		
Grimaldi et al., in press		140	550	856	2551	306
Eulitz et al., 1995; Diesch et al., 1996	/u/		250	600	2500	
Poeppel et al., 1997		M 100 f 200	M 310 f 370	M 870 f 950	M 2250 f 2670	
Obleser et al., 2004a		M 123 f 223	M 318 f 417	M 1357 f 1731	M 1980 f 2627	M 1039 f 1314
Obleser et al., 2004b		Min 112 max 118	Min 231 max 256	Min 522 max 645	Min 2117 max 2292	Min 266 max 415
Scharinger et al., 2011a			350	800	2900	
Grimaldi et al., in press		130	310	660	2437	349
Diesch et al., 1996	/ø/		350	1400	2500	
Poeppel et al., 1997		M 100 f 200	M 310 f 370	M 870 f 950	M 2250 f 2670	M 100 f 200
Obleser et al., 2004a		M 123 f 223	M 318 f 417	M 1357 f 1731	M 1980 f 2627	M 1039 f 1314
Obleser et al., 2004b		Min 108 max 125	Min 301 max 325	Min 133 max 1447	Min 1945 max 2079	Min 981 max 1142
Obleser et al., 2004b	/y/	Min 115 max 144	Min 238 max 248	Min 1516 max 1769	Min 1987 max 2097	Min 1275 max 1528
Scharinger et al., 2011a			300	2000	2600	
Scharinger et al., 2011a	/ɯ/		350	1800	2600	

*The tokens of the vowels that have been used in the experimental protocols are generally spoken by native male speakers with some exceptions reported in the table: (m) stands for male voice and (f) for female voice. Empty cells mean that the study has not tested that vowel category*.

However, the studies on natural and large sets of vowels lead to quite different conclusions. For example, Scharinger et al. (2011a) used natural stimuli investigating the entire Turkish vowel system that symmetrically distinguishes between high/non-high ([i, ɯ, y, u]/[ɛ, ɑ, œ, ɔ]), unrounded front/back ([i, ɛ]/[ɯ, ɑ]) and rounded front/back ([y, œ]/[u, ɔ]) vowels (cf. Table 2). By means of a mixed model statistical approach, they tested whether the N1m complex was better accounted for by acoustic gradient predictors (acoustic model) or by distinctive features oppositions (feature model). Results for the acoustic model revealed that N1m increased with decreasing F1 and F2 values: i.e., the largest N1m amplitudes were found for high back vowels (e.g., [u]). In contrast, N1m amplitudes decreased with decreasing F3 values. Intriguingly, the feature model fitted the data better than the acoustic model and resulted in larger N1m amplitudes to high vowels (e.g., [u]) than to non-high vowels (e.g., [ɑ]). In addition, rounded back vowels (e.g., [u]) elicited higher amplitudes than rounded front vowels (e.g., [y]), whereas unrounded vowels showed the opposite pattern. Interestingly, similar results have been recently found by Grimaldi et al. (in press) in an EEG study exploring the N1 component. They investigated the Salento Italian (SI) vowel system, i.e., [i, ɛ, a, ɔ, u], where [i, u] are high and [ɛ, a, ɔ] non-high vowels. Accordingly, they found that high vowels elicited larger amplitude than non-high vowels showing a categorical effect for phonological patterns. Overall, these findings led to the conclusion that the processing of a vowel system did not rely on the full available acoustic frequency gradients; rather it relies on the abstract representation of articulatory plans and acoustic output, i.e., the binary oppositions of distinctive features.

With the aim of addressing the same issue from a different perspective, Scharinger et al. (2012) explored the American English vowels [æ], [ɛ], and [i] using the N1m and the MMNm components. According to the phonological model developed by Lahiri and Reetz (2002), the mid vowel [ɛ], which is neither high nor low, is entirely underspecified for height, as it is clearly collocated in the mid of the acoustic-articulatory space between low [æ] and high [i]. Within the MMNm framework (cf. Sections Introduction and The N1/N1m Wave and Its MMN/MMNm Counterpart), the mid vowel [ɛ], preceded by the low vowel [æ] (fully specified for low), should elicit a larger MMNm response than in the reverse case, that is, if the low vowel [æ] is preceded by the mid vowel [ɛ]. In the former (but not the latter) case, the standard generates a strong prediction regarding its tongue height specification that is then violated by the deviant. This is because a fully specified low or high vowel in standard position should generate a strong expectation regarding tongue height specification that might be violated if the deviant to this standard sequence is an underspecified mid vowel. Further, assuming a low vowel as a standard ([æ]) and a high vowel as a deviant ([i]) would lead to a featural mismatch between [low] and [high]. This mismatch, however, should be observed in the reverse case as well (Scharinger et al., 2012). The results confirmed the hypothesis both for the N1m and the MMNm. Actually, when deviant [ɛ] was preceded by standard [æ], the N1m amplitude was significantly larger as compared with the N1m elicited by [ɛ] in standard position. In contrast, the deviant [æ] preceded by standard [ɛ] did not elicit a larger N1m compared with the corresponding N1m of standard [æ] and deviant [ɛ]. Large MMNms occurred when the mid vowel [e] was a deviant to the standard [æ]; this result is consistent with less specific representations for mid vowels. MMNms of equal magnitudes were elicited in the high–low comparison, consistent with more specific representations for both high and low vowels. However, evidence for underspecified features was not successively found for labio-velar and palatal glides labial and palato-alveolar fricatives ([awa]-[aja] and [ava]-[aʒa]) (Scharinger et al., 2011b).

As for consonants, the available data are scarce. First evidence was that stop consonants ([b, p, d, t, g, k]) produced higher N1m amplitude than non-stop consonants ([f], [r], [m], [r], [s]) in both hemispheres (Gage et al., 1998) (cf. Section N1m Hemispheric Asymmetries for Vowels, Consonants, and Syllables). Also, the N1m amplitudes seem to vary as a function of the onset of the speech sounds with a higher amplitude for labial [ba] than alveolar [da] as compared to velar [ga] in both hemispheres. Perception of stop consonants relies on cues realized on transitions from the stop to the adjacent vowels: (i) the burst of noise generated after the rapid release of complete closure of the vocal tract (less intense for voiced than for voiceless stops); (ii) the voice-onset-time (VOT), the time lag between the consonantal release and the start of vocal-fold vibration in a following vowel (negative for voiced stops and either zero or positive for voiceless stops); (iii) the fundamental frequency and the first formant values during adjacent vowels, which are lower in proximity to the voiced stop. Thus, the overall difference in amplitude to stops vs. non-stops may be attributed to the acoustic differences in the onset dynamics of these two classes of stimuli. At the same time, it seems that N1m amplitude is sensitive to PoA. Within the class of stop consonants, the N1m peak amplitude resulted also sensitive to the feature Voicing as revealed in the study by Obleser et al. (2006), where four different stops (alveolar-voiced [d], alveolar-unvoiced [t], velar voiced [g], velar-unvoiced [k]), spoken by four different speakers (two males, two females) were investigated. The stimuli were presented in an intelligible format as well as in an unintelligible spectrally inverted format. Peak amplitude revealed that only intelligible voiced consonants yielded the stronger N1m amplitudes than their unvoiced counterparts did.

### Latency for vowels, consonants, and syllables

A recent study (Swink and Stuart, 2012) has compared non-speech vs. speech stimuli and natural vs. synthetic speech stimuli, demonstrating that the N1 latencies are significantly shorter when evoked with the tonal stimulus vs. speech stimuli and for natural vs. synthetic speech. These findings are in line with the initial experiments on vowels, which revealed longer processing for vowels than for tones and, more interestingly, regular patterns in response to the vowel acoustic properties (Eulitz et al., 1995; Diesch et al., 1996; Poeppel et al., 1997). In general, these studies reported that the N1m latency was reliant on the acoustic correlates of speech segments such as F1 frequency. However, an exception was the study of Eulitz et al. (1995), who found that the synthetic vowels [a, æ, i, œ, u] fall into the same latency range. Conversely, Diesch et al. (1996) showed systematic stimulus-specific differences between the latency responses to the synthetic German vowels [a, u, i, æ, ø]: i.e., the [a] and [æ] vowels, with high F1 values, evoked shorter latency than [u]. Poeppel et al. (1997) replicated this result: among the synthetic vowels [a], [i] and [u], the vowel [a] (F1 700 Hz) evoked an earlier latency than [u] and [i]. The authors attributed this latency advantage of [a] to the proximity of its first two formants to 1 kHz (see also Roberts et al., 2000). The spectral dominance of the first formant position for vowel encoding was confirmed by Obleser et al. (2003a) reporting faster N1m responses to the German vowel [a] than [e] and [i] (Table 2). However, the N1m variations appeared to be most pronounced in the range from 100 Hz to 1 kHz, i.e., the range of the F1 distributions, failing, once again, to establish whether the N1m shifts reflected spectral or rather phonetic processes.

Subsequent N1m research attempted to explain speech encoding by referring to mere discrete featural variables rather than to mere acoustic properties. For example, Roberts et al. (2004) analyzed the timing of the auditory responses to a continuum of synthesized vowels varying along the F1 dimension with clear exemplars of [u] and [a]. It was found that the N1m latency was dependent on the categorical membership. Therefore, they argued that the physical stimulus properties and the F1 in particular, dominated only the N1m latency for tokens that were not members of a distinct language category. Yet, the N1m latency hemispheric differences among German vowels [a], [e] and [i] were interpreted as putative evidence of a special tonochronic processing for speech encoding (Eulitz et al., 2004). In 10 MEG recordings on a single subject, the authors found that phonologically closer vowels, e.g., [e]-[i], did not affect the temporal modulations of the N1m responses. Later studies on natural vowels confirmed such assumptions. Obleser et al. (2004a) found that the back vowel [o] consistently elicited later responses than the front vowel [ø] (on average 5 ms). Again, but this time with a large set of German vowels ([i, e, ø, y, a, o, u]), Obleser et al. (2004b) showed that the back vowels (e.g., [o]-[u]) peaked later than the front vowels (e.g., [e]-[i]) and that high front vowel (e.g., [i]) peaked later than a non-high front vowel (e.g., [e]). Note that front unrounded [e]-[i], front rounded [y]-[ø] and back rounded [o]-[u] showed very similar F1 Hz values and that although the vowel [u] elicited the latest N1m response, the vowels [i] and [y] did not. According to the MEG results of Obleser et al. (2004a,b), the EEG study by Grimaldi et al. (in press) revealed a later N1 processing for the SI vowels marked by the features [+back] (i.e., [a], [ɔ], and [u]) than their [-back] counterparts (i.e., [i], [ɛ]). Such pattern was found in the late N1 activities, at about 150 ms from stimulus onset, and was interpreted assuming that the formant frequencies alone cannot account for the auditory processing of vowels when binary oppositions of distinctive features play a relevant role in contrasting phonemes within a linguistic system. The mapping rules seem to proceed in a different way when testing the large set of the Turkish vowel system (Scharinger et al., 2011a). This study found that back vowels (e.g., [u]) were earlier than front vowels (e.g., [y]), and that the features Height and Round affected the timing neuronal strategies resulting in later responses to high (e.g., [i]) than non-high (e.g., [ɑ]) vowels and in faster N1m to unrounded vowels (e.g., [m]) than to the rounded counterparts (e.g., [u]).

The N1m latency was found to be involved for PoA and Voice in consonants. Obleser et al. (2003b) investigated naturally spoken CV German syllables consisting of stop consonants with different PoAs—[b] labial, [d] alveolar and [g] velar—and front rounded [ø] or back rounded [o]: i.e., [bø], [dø], [gø] and [bo], [do], [go]. The N1m peak latency highlighted that the velar-back rounded CV syllable [go] elicited a later response than other syllables, confirming the critical role of PoA for temporal coding in human speech recognition (Roberts et al., 2000; Gage et al., 2002; Obleser et al., 2004b). The combination of back stop and back vowel features also seemed to delay the N1m peak latency. According to the authors, this suggests that the assimilatory effect of a back vowel is very influential on a back consonant like [g]. The low formant frequencies resulting from the presence of the place feature back in both consonants and vowels as in [go], may prolong the temporal integration process reflected in the N1m responses (cf. Sections Amplitudes for Vowels and Consonants and N1m Hemispheric Asymmetries for Vowels, Consonants, and Syllables).

The features PoA and Voice affected the N1m latencies when isolated natural consonants were compared to the unintelligible counterparts (Obleser et al., 2006). The N1m to alveolar consonants [d] and [t] peaked earlier than responses to velar consonants [g] and [k], and voiced consonants [d] and [g] peaked later as compared to voiceless consonants [t] and [k]. However, these temporal strategies were not modulated by intelligibility. The authors, thus, proposed that the latency changes were mainly driven by the spectral (Place, spectral peak of [d-t] vs. [g-k]) and temporal (Voicing, voice onset time, [d-g] vs. [t-k]) features of the stimuli.

### The N1m/N1 generators for vowels, consonants, and syllables

Most of the available studies have described the existence of speech coding patterns by adopting the dipole approach for modeling the N1m patterns and by observing the spatial arrangement into the brain. Early investigations adopted a strongly localizationist perspective indicating the core auditory regions as the most responsive areas to the physical acoustic properties (Diesch et al., 1996; Poeppel et al., 1996). Moreover, a commonplace assumption is that the sound salient features for the phonological encoding drive the displacement of the N1m generators, which define specific arrangements (maps) on the cortical sheet.

A number of studies measuring the absolute Euclidean distance between the N1m ECDs showed that large spectral and phonological dissimilarities of vowels are preserved at a cortical level (see Table 3). Obleser et al. (2003a) tested the three synthetic German vowels [a], [e], [i] and reported large cortical distances between the most acoustically dissimilar vowels in F2-F1 space such as [a]-[i] when compared to the most similar vowels [e]-[i]. The study of Mäkelä et al. (2003) with Finnish vowels [a], [o] and [u] reported the same results. Shestakova et al. (2004) testing the Russian vowels [a], [u], [i] confirmed the idea that vowels close in the F2-F1 dimension were close in the Perisylvian regions: [a]-[i] and [a]-[u] showed larger distances than [i]-[u]. Moreover, some authors demonstrated that the vowel cortical differences were preserved even when taking into account the specification for phonological features: that is, it is likely that the absence or presence of one or more distinctive features affect the N1m location. For example, Obleser et al. (2004b) found that German front vowels [e]-[i] and front–rounded vowels [ø]-[y] differing in only one place feature were more closely collocated within the anterior parts of the STG than front vowels [e]-[i] and back–rounded vowels [o]-[u] differing in two features. Again, the work of Scharinger et al. (2011a) on Turkish vowels showed that vowels differing for Place and Round, e.g., [a-œ], displayed larger distances than vowels differing for Round only, e.g., [a-o] (Table 3).

**Table 3:** **Results for the Absolute ECD distances and for the significant effects driving the dipole location along the medial-lateral, anterior-posterior, and inferior-superior dimensions**.

**References**	**Sj**	**Language**	**Vowel**	**Absolute ECD Distance (d)**	**Med./Lat**.	**Ant./Post**.	**Inf./Sup**.
Eulitz et al., 1995	11	Synthetic	[a, i, u, æ, œ]	–	–	–	–
Diesch and Luce, 1997	11	Synthetic	[a, æ, u, ø, i]	–	F1	–	–
Poeppel et al., 1997	6	Synthetic	[a, i, u]	–	–	–	–
Mäkelä et al., 2003	10	Finnish synthetic	[a-o-u]	[a-o] < (d) [a-u]—left hemisphere	–	F1 and F2	–
Obleser et al., 2003a	11	German synthetic	[a-e-i]	[a-i] > (d) [e- < i]	–	–	F1 and Height
Shestakova et al., 2004	11	Russian natural	[a-u-i]	[a-i], [a-u] > (d) [i-u]	–	–	F2-F1
Eulitz et al., 2004	1	German synthetic	[a-e-i]	[a-i] < (d) [e-i]	F2-F1	–	–
Obleser et al., 2004a	14	German natural	[ø-o]	–	Place	–	–
Obleser et al., 2004b	20	German natural	[a-e-i-ø-y-o-u]	[e-i], [o-u] > (d) [e-i], [ø-y] [a-i] > (d) [e-i]	–	Place	–
Scharinger et al., 2011a	12	Turkish natural	[i-ɯ-ɛ-ɑ-y-œ-ɔ-u]	[a-œ] > (d) [a-o] [u-ɛ] > (d) [u-i]	F2 Round	Place	Round and Height in front vowels
Scharinger et al., 2012	14	American English natural	[æ. ɛ, i]	[œ-i] > (d) [œ-ɛ]	–	Height	Height—left hemisphere
Kuriki et al., 1995	6	Synthetic	[na], [ka], [ha], [a]	–	Onset of the low-amplitude high-frequency consonant	–	–
Obleser et al., 2003b	16	Natural German	[bø], [dø], [gø] [bo], [do], [go]	–	–	Place	–
Obleser et al., 2006	19	German natural	[d], [t], [g], [k]	–	Place	Place	Place
Scharinger et al., 2011b, Experiment 1	13	American English natural	[aja], [awa]	–	–	Place—Labial—(as deviant)–left hemisphere	–
Scharinger et al., 2011b, Experiment 2	15	American English natural	[ava] [aʒa]	–	–	Place—Labial (as deviant and as standard)—left hemisphere	–
Scharinger et al., 2011b, Combined analysis Experiment 1 and 2			[aja], [awa], [ava] [aʒa]	–	–	Place—Labial	–

*The labeled Sj reports the number of the subjects that were submitted to statistical analysis. The symbol (–) means that the results did not reach statistically significance. Empty cells in column labeled Absolute ECD distance (d), means that the study has not performed that kind of analysis. All studies listed report results from both hemispheres, unless otherwise noted*.

**Figure 6:**
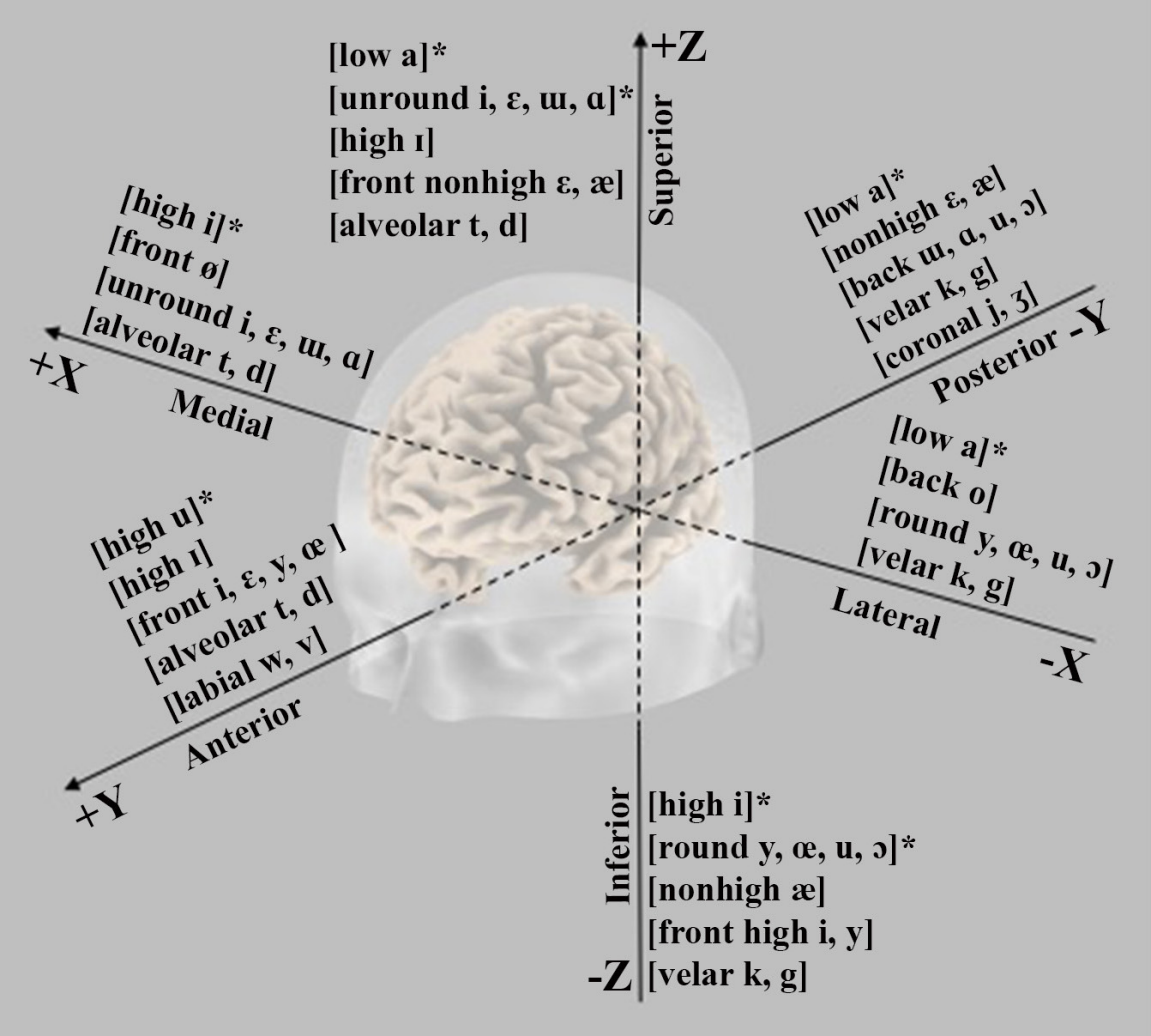
**Graphical representation of the main trends emerging from the N1m ECD analysis along the three-dimensional spaces slicing human brain in lateral-medial (***x***), anterior-posterior (***y***), and inferior-superior axis (***z***)**. The symbol (^*^) indicates that the topographical gradient was explained in terms of acoustics effects rather than of featural variables.

Another perspective commonly used for investigating the existence of a putative acoustic–phonetic neural map is the analysis of the ECD gradients in the 3D source space within the temporal lobes of the auditory cortex, i.e., lateral-medial, anterior-posterior, and inferior-superior (Table 3 and Figure 6). The lateral-medial axis is the plane in which a tonotopic structure was early discovered. Pioneering N1 studies revealed that stimuli with high frequencies were deeper (more medial) in the brain than stimuli with low frequencies (Pantev et al., 1995; Woods, 1995). Vowel studies rarely confirmed the same patterns and when they do the results are often contradictory. Diesch and Luce (1997) found medial locations for ECDs exerted by vowels with F1 high frequencies. Eulitz et al. (2004) found that the German vowel [i] with large spectral F2-F1 distance, was more medial than vowels with close formants peaks (e.g., [a]). Further studies described a broad cortical vowel distinction according to different phonological patterns. Obleser et al. (2004a) observed that the German back vowel [o] was lateral to the front vowel [ø]. On their part, Scharinger et al. (2011a) demonstrated that the dipole movements were more affected by phonological features than by acoustic properties and found a gradient for Round along this plane: i.e., rounded vowels ([y, œ, u, ɔ]) were at more lateral positions than unrounded vowels ([i, ɛ, ɯ, ɑ]). Moreover, a MEG study into monosyllabic speech sounds (vowel [a], nasal-consonant vowel [na], plosive-consonant vowel [ka], and fricative-consonant vowel [ha]) showed that along the mediolateral axis the spatiotemporal shifts were specific to the consonants. The plosive and fricative response sources shifted laterally with latency, but the vowel response source shifted medially. The nasal response source did not show significant shifts (Kuriki et al., 1995). Again, Obleser et al. (2006) showed that the N1 responses to intelligible front consonants [d] and [t] originated from medial locations.

Along the anterior-posterior plane, the first probes with non-speech stimuli revealed that the high frequencies had anterior N1m dipoles (Langner et al., 1997; Diesch and Luce, 2000). In speech encoding, such gradients were shown to be sensitive to the F1 and F2 values. Mäkelä et al. (2003) observed that Russian vowels with the lowest ([u]) and highest ([a]) F1 and F2 were located anteriorly and posteriorly, respectively, and that the area activated by the intermediate vowel ([o]) was located between these two, probably due to the inhibitory hypothesis (cf. Section Amplitudes for Vowels and Consonants). This result, however, might be also interpreted in relation to the different F1 values of vowels: so that, the lowest F1 ([u]) and highest F1 ([a]), differentiating high and non-high vowels, generated opposite centers of activation with the intermediate F1 values ([o]) being collocated between the two extremes. Actually, Scharinger et al. (2012) found a broad distinction between high ([i]) and non-high ([ɛ], [æ]) American English vowels: [i] elicited a dipole that was located 7.5 millimeters (mm) more anterior to the dipole of either [ɛ], [æ]. Studies with a larger set of stimuli, as in the case of the works of Obleser et al. (2004b) and Scharinger et al. (2011a), revealed that the N1m source locations correlated with the F2 values directly dependent by the Place phonological feature. In the German vowel system, back vowels (e.g., [o], [u], and [a]) appeared at more posterior locations than front vowels (e.g., [e], [i]), whereas coronal and labial vowels did not differ significantly. Yet, in the Turkish system, front [i, ɛ, y, œ] were more anterior than back [ɯ, ɑ, u, ɔ] (Scharinger et al., 2011a). Kuriki et al. (1995) revealed that the source of consonant sounds (i.e., fricative response) was more posterior, on average, than the plosive response sources, the vowel and the nasal response sources. The anterior-posterior dimension mirrored also the spectrotemporal properties associated with the PoA changes within CV syllables ([bø], [dø], [gø] – [bo], [do], [go]) as shown in the study by Obleser et al. (2003b). The authors found that the differences in N1m source location were dependent on the PoA of the vowel but independent of the different syllable onsets. Due that the formant transitions in coarticulated CV syllables bear information about the adjacent vowel (Ohala, 1993), the front vowel [ø] elicited activity anterior to dorsal vowel [o]. Moreover, the authors concluded that the mapping for PoA could be dependent on intelligibility. In a subsequent study, Obleser et al. (2006) showed that the N1m sources of the intelligibility alveolar [d] and [t] were more anterior than velar [k] and [g] irrespective of the voicing feature of the stimuli. When labial and coronal consonants were compared (as in the couple [awa]-[aja] and [ava]-[aʒa], respectively (Scharinger et al., 2011b), labials elicited dipoles with more anterior locations than coronals. This spatial location was independent of manner of articulation: anterior–posterior locations did not differ between [w] and [v] or [j] and [ʒ]. A statistical model comparison showed that although the F2-F1 ratio was the best predictor for an acoustic model, a model built on the additional fixed effect place (labial/coronal) provided a better fit to the location data along the anterior/posterior axis. This might be interpreted as evidence for top-down categorical effects on the acoustically driven dipole location in auditory cortex.

Few of the evaluated N1m studies reported significant results along the inferior-superior axis. By comparing ECDs to Russian [a], [u] and [i], Shestakova et al. (2004) found that the ECD for [a] was superior to the ECD for [i]; additionally, [u] appeared between [a] and [i] but it did not significantly differ from them. Authors interpreted their data as an effect of the spectral differences at the light of the inhibitory effect (cf. Section Amplitudes for Vowels and Consonants). The work by Eulitz et al. (2004) in the single study-case with the three synthetic German vowels [a], [e] and [i] led to similar conclusions. Obleser et al. (2003a) tested the same synthetic vowels (see Table 3) but they did not find differences in the ECDs location to [a] and [i], rather they found a superior location for [a] than for [e] and [i] as effect of F1 and Height. Conversely, Scharinger et al. (2012) revealed that the dipoles for the high [i] were approximately 7 mm more superior to the dipoles for the low [æ], whereas the locations between [i] and [ɛ] and between [ɛ] and [æ] did not differ. Finally, Scharinger et al. (2011a) revealed a Round effect on the dipole locations, so that rounded vowels, which are acoustically marked by low F2 frequencies, were located at more inferior locations than dipoles to non-round vowels. However, when this effect was investigated for Front and Back vowels separately, the authors stated that the F1 and the related Height effects were, once again, the guiding rules for the cortical segregation within Front vowels only. As for consonants, Obleser et al. (2006) showed front consonants (e.g., [d, t]) a more superior location than back counterparts ([k, g]).

### Summary

It is hypothesized that N1/N1m responses evoked with the non-speech tokens differ from those recorded with the speech tokens that show stronger amplitude and longer latency. Initial findings focusing on vowel discrimination tasks suggest that their representation is mainly guided by the spectral relations of frequencies rather than by abstract, linguistically relevant categories (with a potential reference to distinctive features); thus, it is reported that vowels with large F2-F1 distance (e.g., [i], [u]) elicit larger amplitudes than vowels with close F2-F1 formants peaks (e.g., [a]). When sets of vowels are compared, broad indications of phoneme distinction are associated to the processing of featural variables: for example, high (e.g., [i, u]) show larger amplitudes than non-high vowels, and rounded back vowels (e.g., [u, ɔ]) generate higher negativities than rounded front vowels, whereas unrounded vowels show the reverse patterns. However, few studies were successful in reporting amplitude data probably for practical reasons dealing with the MEG equipment (e.g., the subjects' head-placement in the scanner) or with the procedures of data analysis (e.g., the computation of the root mean square that normalizes the differences across participants) (Gevins et al., 1994). Furthermore, the N1m latency appears to be mainly related to the F1: i.e., high F1 values (e.g., [a] and [æ]) evoke shorter latency than low F1 values (e.g., [i] and [u]). Yet, works focusing on entire phonological systems highlight that the N1m/N1 changes are related to the abstract processing of phonological features, although still tentatively: high vowels peak later than non-high vowels; additionally, back vowels elicit later responses than front ones. For the Turkish vowel system, however, back vowels seem to be processed earlier than front vowels. As for consonants, stable evidence pertains to stops segments: stops produce higher amplitudes than non-stop counterparts (because of the acoustic differences in the onset dynamics of these two classes of stimuli), and, within the class of stop consonants, the voiced consonants produce stronger amplitudes than the unvoiced ones. On the other hand, PoA and Voice seem to affect also the time processing of alveolars that peak earlier than velars, and of voiced consonants that peak later than voiceless.

In line with the amplitude and latency findings, the absolute Euclidean distances between the representational centers of vowels (cf. Table 3) reveal that the most dissimilar vowels in the F2-F1 space tend to generate larger cortical distances than the most similar ones. Also, some studies report that the vowels marking by one distinctive feature are closer than vowels maximally different for two or more features. The abstract representation of vowels emerges more clearly for the relative distances along the Talairach axes. Actually, the N1m dipoles appear dependent on both spectro-temporal cues and phonetic features. In particular, (i) the lateral-medial axis showed medial locations for sounds with F1 high values and large F2-F1 distance and lateral positions for back or rounded vowels relative to sources of front or unrounded vowels; (ii) the anterior-posterior plane is responsive to the F1 and F2 values associated with Height and PoA: in the first case, differentiating high (more anterior) from non-high vowels and in the second case differentiating front (more posterior) from back vowels, as well alveolars and labials (more anterior) from velars and coronals; (iii) the inferior-superior axis shows sensitivity to F1 and Height, but this finding does not seem solid because of different kinds of stimulus among the studies. Yet, the sources of rounded vowels turn out to be inferior to those of non-round vowels and, for consonants, alveolars are superior to velars (cf. Figure 6).

## N1m hemispheric asymmetries for vowels, consonants, and syllables

As noted above (Section Introduction), a key issue in speech perception processing is whether the functional organization of the auditory cortex entails symmetric or asymmetric hierarchical processing mechanisms. Studies of speech and non-speech processing in adults, indicating a rightward bias for non-speech processing: Diesch et al. (1996) found that the cortical separation of pure sine tones and vowels was more distinct in the left than in the right hemisphere. A study of Parviainen et al. (2005) comparing speech (e.g., vowels ([a, u] and CV syllables [pa, ka]) vs. matched non-speech signals found N1m important differences between the stimulus type in the left regions, exclusively. However, in some studies, hemispheric data was not reported (Diesch and Luce, 1997, 2000), whereas many others yielded evidence for the bilateral activation of the auditory cortex (Diesch et al., 1996; Poeppel et al., 1997; Obleser et al., 2003a,b, 2004b; Shestakova et al., 2004). Otherwise, some recent MEG studies have showed intriguing N1m latency modulations associated with left-lateralized processing of phonemes For example, Eulitz et al. (2004) found that the vowel [e] was close to the [i] in the left hemisphere while it was close to [a] in the right hemisphere. That is, only in the left hemisphere the most phonologically closest stimuli showed a temporally coherent behavior. In the study of Mäkelä et al. (2003), the three vowels [a, o, u] activated separate areas in the auditory cortex of the left hemisphere only. Along this line, by exploring two levels of statistical analysis (acoustic and phonological) on sensor and source-space data, Scharinger et al. (2011a) showed that the acoustics-based variables were better predictors for the right hemisphere whereas the phonological-based variables were better predictors for the left, although only for some vowel comparisons. A recent EEG investigation of the SI vowel system (Grimaldi et al., in press) that showed two temporally and hierarchically different N1 components (cf. Section Latency for Vowels, Consonants, and Syllables), the first component peaked bilaterally at 125–135 ms on medial electrodes around the vertex (in the A1, BA 41), and the second peaked at 145–155 ms with a clear leftward asymmetry (in the STG, BA 22). giving for the first time evidence for different N1 waves sub-components as hypothesized by studies with non-speech stimuli (Näätänen and Picton, 1987; Woods, 1995).

The left and right hemispheres appear to be differently involved when we look at the perceptual processing of consonants (C or CV), although the works are yet limited. A very early EEG evidence was that the left-hemisphere N1 peak was larger during the discrimination of stop consonants ([ba], [da]) than during the discrimination of the fundamental frequency, suggesting specific auditory processing for these kinds of sounds (Wood et al., 1971). Subsequent MEG studies better clarify this issue. Kuriki et al. (1995) used N1m to examine the response to different types of speech sounds (vowel [a], nasal consonants [na], velar stop-consonants [ka], and fricative consonant [ha]). Yet, they showed that the left auditory regions were sensitive to different types of sound: consonants containing high-frequency noise at their onset (fricative and plosive) are spatially different from vowel sounds. Poeppel et al. (1996) found hemispheric asymmetries to be task dependent: by using synthesized stop consonant CV syllables ([bæ, pæ, dæ, tæ]), they recorded longer N1m latencies in the left hemisphere for stop consonant syllables than in the right only when attention was required by listeners, raising the possibility that the two hemispheres are treating speech stimuli differently in some way. In that study, however, only stop consonants were used and the paradigm tested selective attention, so there is no information about how speech sounds with different onset dynamics may be processed in the two hemispheres. Finally, Gage et al. (1998) compared stop ([b, p, d, t, g, k]) and non-stop consonants ([f, r, m, r, s]), providing evidence for latency hemispheric asymmetries: latency of the N1m component for non-stops was longer in the right than in the left hemisphere, probably due to the differences in the onset dynamics of these two classes of stimuli (stops contain more energy at the onset than no-stops). Again, hemispheric asymmetry for the N1m latency, but not for its amplitude, was found in a later work of the same research group (Gage et al., 2002). Their data demonstrated a small but consistent latency shift in the right hemisphere as a function of PoA, with longer latency for labial [ba] as compared to alveolar [da] or velar [ga]. Overall, these findings suggest that left and right auditory cortical fields make differential contributions to the spectro-temporal processing of sounds.

To sum up, the N1m studies into vowels find (i) no clear clues of asymmetric processing in left and right auditory cortex (Diesch et al., 1996; Poeppel et al., 1997; Obleser et al., 2003a,b, 2004a; Shestakova et al., 2004); (ii) a large tone-vowels difference in the left hemisphere (Eulitz et al., 1995); (iii) interhemispheric differences in the generation of the N1 latencies (Eulitz et al., 2004); (iv) the activation of distinct areas in the left auditory cortex as a function of the F2-F1 distance (Mäkelä et al., 2003); and (v) evidence for feature-based predictors at support of a leftward vowel processing and for acoustics-based variables for the right hemisphere (Scharinger et al., 2011a). Conversely, the N1m studies into consonants show that the difference for the PoA is preferentially computed in the right auditory cortex (Gage et al., 2002). Specifically, stops are preferentially computed on the left and non-stops on the right auditory cortex (Gage et al., 1998), especially when attention is required by listeners (Poeppel et al., 1996). This is in line with a recent 7T fMRI study that found stronger left lateralized activation for alveolar stops than labials (Specht et al., 2014). Crucially, none of the N1m studies was successful in proving different hemispheric processing for the different VOTs in voiced and voiceless consonants.

On the whole, these data confirm the idea that vowels and consonants characterizing by different time-scales, are treated differently by the auditory perceptual system (Liberman et al., 1967; Pisoni, 1973), although a vowel preference for the left hemisphere—as hypothesized by some neuropsychological studies (Caramazza et al., 2000)—has not been confirmed (see however Obleser et al. (2010) for fMRI data showing different distributed hierarchical organizations of vowels and consonants).

## Phonemotopy, phonemochrony and the other side of the coin

Do the available findings support a direct link between linguistic and neurophysiological primitives? That is, can tonotopy and tonochrony (as mirrored in N1m/N1 patterns) explain the properties of the phoneme computations and representations in term of distinctive features within the auditory cortex? Cumulatively, the reviewed N1 studies suggest that the spatial and temporal codes for speech sounds rely on acoustic-articulatory patterns that affect amplitudes, latencies and the spatial arrangement of neural sources in line with the phonological features hypothesis. However, to find a ubiquitous system for speech-specific processing by means of N1m/N1 is not easy. When we look at available data, it is hard to disambiguate between N1m/N1 evidences suggesting pure acoustic patterns and those indicating abstract phonological features. Actually, the solid evidence is that the acoustic distance between the first two formants of a vowel is preserved in the auditory cortex and is directly reliable in sensor and source data, as found in the mammalian auditory cortex (Shamma, 1985a,b; Ohl and Scheich, 1997). This implies a neuronal sensitivity for interactions between spectral components during vowel discrimination that does not require separate formant representations (in the sense of feature extraction). Nevertheless, some clues of orderly cortical representations of abstract features emerge when couples of vowels are investigated (e.g., Obleser et al., 2004b), or when a phonological system has been tested with an appropriate statistical analysis able to discern different levels of auditory brain operations (Scharinger et al., 2011a). Overall, amplitudes, latencies and spatial gradients in the auditory cortex converge on showing that acoustic-articulatory properties are affected by top-down features such as Height, Place and Round. This seems true also for Voice and Place for consonants (Gage et al., 2002; Obleser et al., 2003b, 2006; Scharinger et al., 2012).

On the other side of the issue, also the MMNm/MMN studies seem to support this view. Perception of vowels or VOT contrasts in the across-category conditions elicit MMNm/MMN amplitudes only for those segments having a contrastive role in the phonological system of listeners (e.g., Näätänen et al., 1997; Sharma and Dorman, 2000). These results suggest that the MMNm/MMN is sensitive to the phonetic category distributions of the subjects' native language. Also, studies on categorical discrimination (generally on consonant continua differing in the duration of VOT) highlighted that listeners are able to perceptually group the acoustic distinct tokens together to form a category. When they perceive a token from the other side of the category boundary, a change is detected as indexed by MMN (e.g., Sharma and Dorman, 1999; Phillips et al., 2000).

Phonemes used to contrastively distinguish lexical meaning may generate non-contrastive variants (i.e., allophones) that regularly appear in specific contexts because of the influence of adjacent vowels or consonants. Kazanina et al. (2006) used a multiple-token design with acoustic varying tokens for each of the stimuli to analyze the sound pair [t–d], which has allophonic status in Korean ([d] occurs between voiced sounds and [t] elsewhere) and a phonemic status in Russian. The results revealed an MMNm response for the Russian listeners but no response for the Korean listeners. The authors concluded that the phonemic representations, but not the allophonic ones, are computed from speech. However, Miglietta et al. (2013) found different MMN patterns. They investigated an allophonic variant generated by a phonological process characterizing southern Salento varieties that raises the stressed low-mid front vowel [ɛ] to its high-mid counterpart [e] when followed by the unstressed high vowel [i]. MMNs were elicited for both the allophonic and phonemic conditions, but a shorter latency was observed for the phonemic vowel pair suggesting a rapid access to contrastive sound properties in the phonological mode. Yet, the discrimination of the allophonic contrast indicates that also allophones—generated by specific rules of the grammar—are part of the knowledge of speakers and then of their memory representations.

Finally, studies investigating whether phonemic representations in the lexicon are underspecified for non-contrastive distinctive features values in the language systems (Section Amplitudes for Vowels and Consonants) showed that MMNm/MMN are elicited only when the deviant stimulus is fully specified by a distinctive feature suggesting that this component is indexing more than just physical properties of the stimulus (e.g., Eulitz et al., 2004; Scharinger et al., 2012; but see Mitterer, 2007 for a different perspective and Monahan et al., 2013 for a detailed discussion of this issue).

To sum up, while the N1m/N1 and MMNm/MMN findings show that the electrophysiological sensitivity to the properties of the stimuli is not exclusively correlated with their physical attributes, but rather with top-down processes associated with phonological categories, they are not so strong to preclude purely acoustic explanations of the auditory activity involved in speech processing: the amplitude data are not sufficient to prove a correlation with phonological patterns and the latency results appear contradictory (cf. Section The N1m/N1 Amplitudes, Latencies, and Source Generators). For example, while Obleser et al. (2004a,b) and Grimaldi et al. (in press) showed that back vowels peaked later than non-back vowels, Scharinger et al. (2011a) revealed the reverse pattern. Finally, only one study with consonants has obtained strong evidence of latency modulations for Place and Voice (Obleser et al., 2006). Although it seems that latencies are probably correlated also with Height and Round features (Scharinger et al., 2011a), it is difficult to establish to what extent the activity involved in speech encoding reflects merely mechanisms of spectro-temporal information extraction or rather of phonological computations. In principle, the variability of the results may be due to the spectral identity of the stimuli applied. Actually, the spectral content for vowels (see Table 2) or the different onsets of sounds for consonants as well as further aspects (e.g., rise-time, sound intensity, or stimulus duration) have a great effect on the early auditory activity (e.g., Näätänen and Picton, 1987; Eulitz et al., 1995). Yet, the different type of stimulus may have a further effect on the auditory patterns (Swink and Stuart, 2012) although supporting evidence for this notion is equivocal. For example, Benson et al. (2001), via fMRI, found differing cortical activations for natural speech vs. synthetic speech, but Uppenkamp et al. (2006), via fMRI again, found an identical pattern of auditory cortical activation. In addition, within the reviewed studies, it is hard to confirm a stable trend also because the main research question did not require careful acoustic matching of the speech and non-speech stimuli, or it was not attempted. So, further studies need, eventually, to support the tonochrony (phonemochrony) principle. The source data—as reflected in the Talairach coordinates—turns out to be problematic, although spatial maps in the auditory cortex show a lateral–medial gradient for Place and Round, an anterior–posterior gradient for Place, and an inferior–superior gradient for Height. Thus, also tonotopy needs to be further investigated. Finally, as we will discuss in the next Section, the ECD approach makes the strong assumption that the N1m sources can be described as a single point (source), therefore it seems to be a restrictive tool for estimating centers of activity over wide-spread auditory area, whereas there is the possibility that the phonemotopic processes involving distinctive features are more fine-grained (especially when phonemes are not in isolation but within syllables and words).

What remains to evaluate is whether early auditory indexes are traceable for the hemispheric processing of vowels and consonants.

## In the left, in the right or in both sides?

In general, focusing on neural specializations for the properties that make salient the sounds of languages may provide an informative account of cortical events that underlie speech perception. Data for hemispheric differences specifically, convey clues about the neuronal sensitivity to both the spectral-temporal properties of auditory inputs and/or the linguistic status of the input; such information may allow us (i) to trace the sequential processing of speech sounds and (ii) to understand if speech signal is processed as speech from the earliest stages of cortical processing and if its origins are interpreted as related to physical or to cognitive features.

The canonical view based on neuropsychological deficit-lesion data assumes the left hemisphere is dominant for speech feature extraction (Geschwind and Levitsky, 1968). Recent models on speech perception suggest a bilateral processing of speech sounds, at least on the initial perceptual level (Hickok and Poeppel, 2007; Specht, 2013; but see Scott and McGettigan, 2013). Moreover, these models maintain the classical view of a predominant function of the left temporal lobe in processing and decoding of speech sounds, but only when a higher hierarchical level of perceptive process is required. At the same time, animal studies (Ohl and Scheich, 2005; Rauschecker and Scott, 2009; Perez et al., 2013) as well as neuroimaging studies in humans (Price, 2012) propose that auditory cortices process different aspects of speech signal and that the ventral areas are implied in acoustic processing while the dorsal areas are in phonological decoding. According to the asymmetric sampling in time (AST) theory (Poeppel, 2003; Boemio et al., 2005), the left auditory cortex appears to optimally process repeated auditory stimuli of 25–50 ms duration (correlating with segmental information), whereas the right auditory cortex appears to optimally process auditory stimuli with a 200–300 ms duration (correlating with syllabic and tonal information). A recent study into oscillatory rhythm seems to confirm this picture in showing that incoming speech signals are asymmetrically transformed into intrinsic oscillations within the auditory cortices: the left auditory cortex shows a large activity in the low gamma frequency band (25–35 Hz), while the right cortex demonstrated more activity in the theta frequency band (4–8 Hz) (Giraud and Poeppel, 2012).

In line with this picture, it is reasonable to hypothesize that the two hemispheres hierarchically process the properties of vowels, consonants, and syllables in different ways. As for vowels, it seems that A1 is involved in the analysis of spectro-temporal properties of sound bilaterally, and that left STG and STS are prevalently (although not exclusively) recruited for the computation and representation of abstract phonological patterns. Turning to consonants, it is argued that CV syllables with different VOTs and PoAs are differently processed by the auditory cortices probably for their different spectro-temporal characteristics (cf. Section Amplitudes for Vowels and Consonants). For example, if the left auditory cortex works on very short auditory segments, it might automatically parse the consonant and the vowel in voiced stops (with short VOT from 0 to 30 ms), whereas the right auditory cortex might be superior in processing voiceless stops (with long VOT from 60 to 110 ms). This, of course, would result in the time-locked N1m latencies and generators.

In N1m studies, when vowels are investigated, neither constant evidence for speech-specific left lateralization nor for hierarchical asymmetries between the ventral and dorsal auditory areas have been found (cf. Section N1m Hemispheric Asymmetries for Vowels, Consonants, and Syllables). Moreover, the dipole origins are not generally reported. When they are identified, the supratemporal plane—an area that includes the A1 and the secondary auditory cortex (Diesch et al., 1996; Poeppel et al., 1997; Obleser et al., 2003a)—the planum temporale (Obleser et al., 2004a) or the area around the STS (Eulitz et al., 2004) are suggested as the centers of the speech processing (cf. Figure 1). On the contrary, functional N1m asymmetries clearly turn up with consonant sounds.

These findings are surprising, at least to some extent, as MEG is thought to be well suited to investigate lateralized phenomena for speech, language, and pitch (Lütkenhöner and Poeppel, 2011). The failure to find hemispheric distribution for the computation and representation of vowels probably has different explanations. First, the variability of the stimulus materials: most of the studies tested semi-synthetic and synthetic stimuli (cf. Table 1) that represent an approximation of natural speech, but, importantly, vowels and consonants were generally presented in isolation: i.e., in a condition that is far removed from natural speech. In fact, it has been suggested that an increasing left lateralization appears to be related to the gradual increasing of multiple levels of computation and representation for complex forms when phonemes are structured within words or pseudowords and stored representation of speech sounds are required (Shtyrov et al., 2005; Hickok and Poeppel, 2007; DeWitt and Rauschecker, 2012). In view of this, future studies should investigate the computation and representation of phoneme processing recurring to words and pseudowords at least.

Finally, we have already noted (Section MEG in Short) that MEG is relatively insensitive to radial sources (generally flowing in secondary auditory areas, as in STG), whereas EEG is sensitive to both tangential and radial sources (Cohen and Halgren, 2003; Ahlfors et al., 2010). Thus, given the relevant role of non-primary auditory regions in speech processing (Price, 2012), one could argue that MEG alone is not powerful enough to explain the origins of the N1m/N1 events and to fully explore the organization of auditory streams. Actually, it is well known that the N1m/N1 is not a unitary event (Näätänen and Picton, 1987; Woods, 1995) and that mirrors the contribution of multiple generators that are likely associated with different types of auditory information coding resulting in different components (cf. Section The N1/N1m Wave and Its MMN/MMNm Counterpart). Among them, the first two probably describe the earliest auditory activity, i.e., the so called N1'/P90 and N1c (Woods, 1995): the former is maximally recorded from the fronto-central scalp, peaks between 85 and 110 ms, and it is generated by tangentially orientated currents in A1; the later may be recorded at mid-temporal electrodes, peaking about 130–170 ms, and is generated by radially oriented neuronal dipole sources probably located in the STG (Wolpaw and Penry, 1975; Picton et al., 1978; Wood and Wolpaw, 1982). This means that a single dipole may not be sufficient to describe in detail what happens during the underlying activations. Intriguingly, a recent EEG study of the entire SI vowel system (cf. Section N1m Hemispheric Asymmetries for Vowels, Consonants, and Syllables) showed two temporally distinct N1 dipoles (Grimaldi et al., in press), the first in the A1 (BA 41), bilaterally, and the second toward the ventral areas of the STG (BA22) showing a gradual leftward gradient. These data support the model discussed above, suggesting that A1 is bilaterally involved in the first stages of vowel processing, i.e., in the identification of main properties of sub-lexical patterns, while STG is recruited in the second stage of processing with a left-hemisphere dominance, i.e., when abstract representations of phonological features are necessary to contrastively categorize speech sounds. In addition, Bidelman et al. (2013) recorded the EEG activity generated at both cortical and subcortical levels of the auditory pathway for the vowel continuum [u-a]. They found that activity from the brainstem mirrors properties of the speech waveform with remarkable fidelity, whereas patterns of late cortical-evoked activity reflect distinct perceptual categories accordant to the abstract phonetic speech boundaries heard by listeners. Again, when the EEG N1 wave is used to investigate voiced ([ba]) and voiceless ([pa]) stops a clear left lateralized component emerges for voiced stop with a shorter VOT (Giraud et al., 2005, 2008; see however Frye et al., 2007). This result was also replicated by Obleser et al. (2007), with an event related fMRI investigation, who found that voiced sounds ([d] or [g]) accounted mostly for the left anterolateral STS activation compared with unvoiced sounds ([t] or [k]).

Paradoxically, after more than two decades of auditory research with MEG, the first evidence—although very preliminary—in confirming neuropsychological and neuroimaging models, comes from EEG investigations. Actually, it has been argued that with the EEG it is possible to dynamically record the electric activity of the brain from more focused regions relative to MEG (Malmivuo et al., 1997). Moreover, to achieve a good generators' estimation starting from the voltage distribution at scalp, the correct sensor locations on the scalp are crucial. This may be critical with MEG, as the experimental subjects are relatively free to position his/her head within the sensor array. Contrary, typical 10/20 EEG montages offer fewer degrees of freedom in that respect (Gevins et al., 1994; Cohen and Halgren, 2003) as well as the 64 to 128 electrodes cups improve measurements spatially. Liu et al. (2002) demonstrated that EEG localization could be even more accurate than MEG localization for the same number of sensors averaged over many source locations and orientations. Crucially, the knowledge reached in software analysis has permitted a solution to the inverse problem also for EEG data thereby improving the accuracy of the volume conductor model of the head (Liu et al., 2002; Grimaldi et al., in press; Manca et al., in press). Thus, in monitoring the hierarchical information processing in which the neural activity flows in both radial and tangential directions, the exclusive use of MEG can be a restrictive factor.

## Future directions and concluding remarks

In this review, we highlighted that the ultimate proof of the existence of speech-specific codes (i.e., phonemotopy and phonemochrony) together with other levels of abstract speech processing cannot be fully described with the available N1m/MMNm studies, whose findings should be considered provisional. The same picture emerges when we look at the sustained field responses (SFs), which showed a certain sensitivity to the spatiotemporal features that are relevant for the phonological encoding. A stable finding is that SFs reliably enhance for vowels and non-periodic vowels as compared to tones (Eulitz et al., 1995; Gutschalk and Uppenkamp, 2011) and that the functional level of these patterns may be directly associated with the formant extraction at the level of the auditory cortex. Yrttiaho et al. (2008) for example, showed enhancement of the SF amplitudes for [e] in contrast to [a] as an effect of the distance between the major spectral peaks of the stimuli. Yet, the dipoles of the SFs show inter-hemispheric asymmetries for vowels, but not for tones, and intra-hemispheric asymmetries when comparing tones to vowels in the speech-dominant left hemisphere. Accordingly, at the moment, we can only speculate that speech maps emerge for unifying acoustic-articulatory information into more abstract featural representations.

On the other hand, the existence of a neuronal network devoted to convert continuous speech signals into discrete representations is not under discussion. In facts, an ECoG study was successful in mapping the entire English vowel and consonant system showing spatiotemporal selectivity of the STG to perceptually relevant aspects of speech sounds (Mesgarani et al., 2014). However, these invasive recording methods have some limitations since data are obtained from abnormal brains and a limited exposed surface of the cortex, precluding an understanding of the dynamics of large-scale inter-regional and hierarchical networks. Thus, these concerns warrant caution in interpreting the ECoG results (Leonard and Chang, 2016).

As we outlined in Section Introduction, a wealth of research suggests that the ERP/ERMF does not simply emerge from evoked, fixed latency–fixed polarity responses that are additive to and independent of ongoing EEG/MEG but by a superposition of ongoing EEG/MEG oscillations that reset their phases in response to sensory input (Bastiaansen et al., 2012; Peelle and Davis, 2012). Such a perspective may provide an alternative way to study the functional neural-network dynamics in speech processing. It has been suggested that a remarkable correspondence between average durations of speech units and the frequency ranges of cortical oscillations exists: phonetic features (mean duration of 20–50 ms) are associated with high gamma (>40 Hz) and beta (15–30 Hz) oscillations; syllables and words (mean duration of 250 ms) with theta (4–8 Hz) oscillations, and sequences of syllables and words embedded within a prosodic phrase (500–2000 ms) with delta oscillations (<3 Hz) (Giraud and Poeppel, 2012; Ghitza, 2013). The basic idea is that there exists a direct relationship between the speech time scales and the time constants underlying neuronal cortical oscillations. This alignment of ongoing oscillatory activity to rhythmic stimuli has been observed in recordings of neural activity in sensory cortex under different attentional conditions (Lakatos et al., 2008; Schroeder and Lakatos, 2009). Thanks to this alignment, the brain may convert speech rhythms into linguistic segments, from phonemes to syllables, words, syntagms, and so on. Thus, continuous neural oscillations may constitute a possible mechanism that leads to online discrete-abstract representation starting from the continuous acoustic flow. In particular, speech onsets trigger cycles of neural encoding at embedded syllabic and phonemic scales through nested theta and gamma oscillations (Giraud and Poeppel, 2012; Arnal et al., 2016).

To date, researchers studying non-speech and speech perception processing have concentrated either on ERPs/ERMFs components or on oscillatory dynamics. However, the relationship between ERPs/ERMFs and oscillatory brain activity has remained elusive. Moreover, whether EEG/MEG oscillations are directly phase reset by the sensory stimulation is not yet well understood (Sauseng et al., 2007). A lot of experimental data illustrate that there exists a meaningful relationship between oscillatory neuronal dynamics, on the one hand, and a wide range of cognitive processes, on the other (Bastiaansen et al., 2012). Visual studies clearly support the hypothesis that the P1–N1 complex is generated by alpha and theta rhythms (Gruber et al., 2005; Klimesch et al., 2007). Due to the limitations highlighted for the N1m studies, it would be very stimulating for future research to focus investigations on what rhythms generate early auditory components such as N1m/N1 and MMNm/MMN for speech and non-speech stimuli and how the phase resetting correlates with the source generators. For example, Haenschel et al. (2000) found that gamma oscillations precede beta 1 oscillations in response to auditory (pure sinusoidal tones) novel stimuli. Palva et al. (2002) showed that for speech sounds ([pa-ka]) there was no difference in the response amplitude between the hemispheres at low (20–45 Hz) gamma frequencies, whereas the amplitude was larger in the right hemisphere for the matched non-speech sounds. These results suggest that evoked gamma-band activity may indeed be sensitive to high-level stimulus properties and may hence reflect the neural representation of speech sounds.

The main advantage in studying oscillations is that the data that are largely lost with the traditional time-locked averaging of single trials used in the ERP/ERMFs approach may be recovered by the analysis of oscillatory dynamics. The analysis methodologies involved in studying oscillatory neuronal dynamics are optimally suited to zoom in on the patterns of synchronization and desynchronization of neuronal activity (Bastiaansen et al., 2012); thus, they provide the necessary means to empirically address issues related to the coupling and uncoupling of functional networks during language processing. This perspective is of crucial importance when we are interested in investigating the online neural decoding of speech processing, because it will permit us to observe phonemes in isolation (the segmental level of analysis) and phonemes within words and sentences (the suprasegmental level of analysis). In parallel, the same perspective may open also a new window to investigate the speech perception-production interface up to now not understood at all (cf. Giraud et al., 2007).

Accordingly, future research into speech sound perception aiming to explore the link between linguistic primitives and neurophysiological primitives should be run bearing in mind the following caveats (further than those discussed in Sections Phonemotopy, Phonemochrony and the Other Side of the coin and In the Left, in the Right or in Both Sides?).

Languages differ widely in their inventories of vowels and consonants (Maddieson and Precoda, 1990; Ladefoged, 2000; de Boer, 2001) and VOT is characterized by timing differences in production and perception across different languages (Lisker and Abramson, 1964): thus, the comparative map of vowel and consonant systems across languages is essential to shed new light on how fine acoustic-articulatory information is neurally warped into more abstract featural representations. Overall, these kind of data—investigated with traditional ERPs/ERMFs components and with oscillatory rhythms—will permit us to better verify amplitude, latency and hemispheric patterns among different systems and to observe to what extent the same 3D coordinates in the auditory cortices mirror the representational processes of phonological features or whether each vowel system, on the basis of peculiar phonological oppositions (along the F1-F2 and F3 dimensions), may optionally select the appropriate Talairach gradients to generate a specific phonemotopic map.

We need to compare the cortical responses to native and non-native phonemes to better test the neuronal sensitivity to acoustic or rather phonological features. If the assumption that the primary auditory areas process acoustic patterns and secondary areas process abstract phonological features holds, it is reasonable to suggest that the perception of non-native phonemes will be bilaterally represented in A1, whereas the secondary regions will fail to generate abstract featural representation of the acoustic parameters. Yet, the usage of non-native sounds will contribute to better verify the 3D representations along the Talairach axes. In parallel, the same data might be useful to observe how—after focused trainings—the phonological representation emerges during the second language acquisition. Again, these data will be very useful to improve the actual models on speech perception.

Phonemotopy, phonemochrony and hierarchical hemispheric asymmetries should be also investigated in pathological populations. For example, cochlear-implant children and normal-hearing children may offer a unique perspective to study how speech computations and representations grow up functionally within the auditory neural network temporally, spatially, and hierarchically distributed. Definitive data in this direction will permit us to refine the theories on speech perception and to prove the validity of the functional-anatomic organization for speech representation in the brain including the tonotopic and tonochronic principles.

## Author contributions

MG conceived the study. MG and ADM designed the study. ADM and MG collected and analyzed the data. MG and ADM wrote the manuscript.

### Conflict of interest statement

The authors declare that the research was conducted in the absence of any commercial or financial relationships that could be construed as a potential conflict of interest.
